# Oral administration of probiotic spore ghosts for efficient attenuation of radiation-induced intestinal injury

**DOI:** 10.1186/s12951-024-02572-8

**Published:** 2024-05-31

**Authors:** Cuixia Zheng, Mengya Niu, Yueyue Kong, Xinxin Liu, Junxiu Li, Xunwei Gong, Xinyuan Ren, Chen Hong, Menghao Yin, Lei Wang

**Affiliations:** 1 School of Pharmaceutical Sciences, Zhengzhou University, Zhengzhou, 450001 China; 2 Translational medicine Center, Huaihe Hospital of Henan University, Kaifeng, 475000 China; 3 Pingyuan Lab, Henan Normal University, Xinxiang, 453007 China; 4 Xinjiang Aksu First People’s Hospital, Akesu, 843000 China; 5 Luoyang Central Hospital Affiliated to Zhengzhou University, Luoyang, 471009 China

**Keywords:** Oral administration, Probiotic spore ghosts, Radioprotection, Intestinal injury

## Abstract

Radiation-induced intestinal injury is the most common side effect during radiotherapy of abdominal or pelvic solid tumors, significantly impacting patients’ quality of life and even resulting in poor prognosis. Until now, oral application of conventional formulations for intestinal radioprotection remains challenging with no preferred method available to mitigate radiation toxicity in small intestine. Our previous study revealed that nanomaterials derived from spore coat of probiotics exhibit superior anti-inflammatory effect and even prevent the progression of cancer. The aim of this work is to determine the radioprotective effect of spore coat (denoted as spore ghosts, SGs) from three clinically approved probiotics (*B.coagulans, B.subtilis* and *B.licheniformis*). All the three SGs exhibit outstanding reactive oxygen species (ROS) scavenging ability and excellent anti-inflammatory effect. Moreover, these SGs can reverse the balance of intestinal flora by inhibiting harmful bacteria and increasing the abundance of *Lactobacillus*. Consequently, administration of SGs significantly reduce radiation-induced intestinal injury by alleviating diarrhea, preventing X-ray induced apoptosis of small intestinal epithelial cells and promoting restoration of barrier integrity in a prophylactic study. Notably, SGs markedly improve weight gain and survival of mice received total abdominal X-ray radiation. This work may provide promising radioprotectants for efficiently attenuating radiation-induced gastrointestinal syndrome and promote the development of new intestinal predilection.

**Supplementary Information:**

The online version contains supplementary material available at https://doi.org/10.1186/s12951-024-02572-8.

## Introduction

Radiotherapy, a common feasible therapy approach, has been extensively used for about 50–60% of cancer patients in clinical settings [1, 2]. However, ionizing radiation inevitably causes damage to surrounding healthy tissues and induces a series of side effects which affect life quality of patients and even reduce efficacy of radiotherapy [3, 4]. Small intestine is one of the most radiation-sensitive organs with large organ volume and is a major injury tissue during abdominal and pelvic radiation therapy [5, 6]. Exposure of small intestine to ionizing radiation can lead to serious gastrointestinal dysfunction, such as vomiting, abdominal pain, diarrhea, infection, perforation, and even death [7]. To date, there are few effective countermeasures for intestinal radioprotection in clinical.

Radiation induced intestinal injury is mainly the results of epithelial cell apoptosis, gut barrier impairment, inflammatory response and intestinal flora imbalance caused by DNA damage and overproduction of reactive oxygen species (ROS) [8, 9]. Amifostine and palifermin are two selective normal tissue radioprotective agents which have been approved by the US Food and Drug Administrator (FDA) [10, 11]. However, amifostine is aimed at reducing side effects of radiotherapy for head/neck cancer and it is contraindicated in patients with liver and kidney dysfunction [12]. Palifermin is mainly used to alleviate radiation induced oral mucositis in patients received radiotherapy [13]. Moreover, both of amifostine and palifermin are small molecular drugs with short half-life and instability in gastrointestinal environment, and they need to be intravenously administered which is not an ideal delivery route for digestive tract disease [14]. Recently, it has reported that some antioxidants, thiols compounds, protein-based pharmaceuticals and bone marrow cell-derived extracellular vesicles exhibit radiation protection potential by eliminating ROS, inhibiting apoptosis and accelerating the restitution of intestinal stem cells [15–18]. However, there are still some challenges to be addressed in their use for intestinal radioprotection, such as intravenous administration and bodywide distribution with varying predilection for tissues [19, 20]. Therefore, it is urgent to develop novel radioprotectants for the prevention of radiation-induced intestinal injury.

Recently, radioprotection based on gut microbiota, such as probiotic therapy and fecal microbiota transplantation, has emerged prevalent as the gut microbiota is closely associated with potential diseases and even to the clear onset of clinical symptoms [14, 21, 22]. It has been reported that restoring the balance of gut microbiota can alleviate radiation-induced intestinal injury in the irradiated animals or patients to some extent [23]. For instance, Guo and co-workers demonstrated that *Lachnospiraceae* and *Enterococcaceae* play a protective role in promoting hematopoiesis and attenuating gastrointestinal damage [24]. Riehl’s group also proved that *Lactobacillus rhamnosus GG* can protect the small intestinal epithelium from radiation injury [25]. Nevertheless, the viability of probiotics and fecal microbiota is affected by gastric acid and ionizing radiation [26–28]. Spores, the dormant life form of probiotics, have been used for drug delivery due to their strong resistance in harsh environment [29, 30]. It is widely known that the high resistance of probiotic spore is from the spore coat which protects nutrient cells from external damage [31, 32]. In previous work, we have provided direct evidence that nanomaterial from spore coat with high tolerance and excellent biocompatibility exhibit a superior anti-inflammatory effect [33]. In addition, this nanomaterial can prevent the occurrence and progression of cancer. These characteristics offer a boundless source of inspiration for radioprotection.

To confirm our speculation, we separated spore coats (denoted as spore ghosts, SGs) from the spore of three clinically approved probiotics (*B.coagulans, B.subtilis* and *B.licheniformis*) for the investigation of intestinal radioprotection (Schem 1). These SGs can scavenge ROS and restore activities of antioxidative enzymes, which effectively restrain X-ray induced apoptosis of small intestinal epithelial cells. Moreover, these probiotic SGs exhibit outstanding anti-inflammatory effect by inhibiting the typical proinflammatory factors such as interleukin 6 (IL 6), tumor-necrosis factor-α (TNF-α) and interleukin-1β (IL-1β) so as to alleviate the inflammatory injury of small intestine. In addition, the three SGs help keep balance of intestinal flora by inhibiting harmful bacteria and boosting proliferation of *Lactobacillus*, so as to promote restoration of barrier integrity. In brief, our findings demonstrate excellent radioprotective effects of SGs by lessening the symptoms of the radiation-induced intestinal injury and improving survival in mice after total abdominal radiation. We envision that the bioinspired material can provide a promising method for the prevention and treatment of radiation-induced intestinal injury.

**Schem 1:**
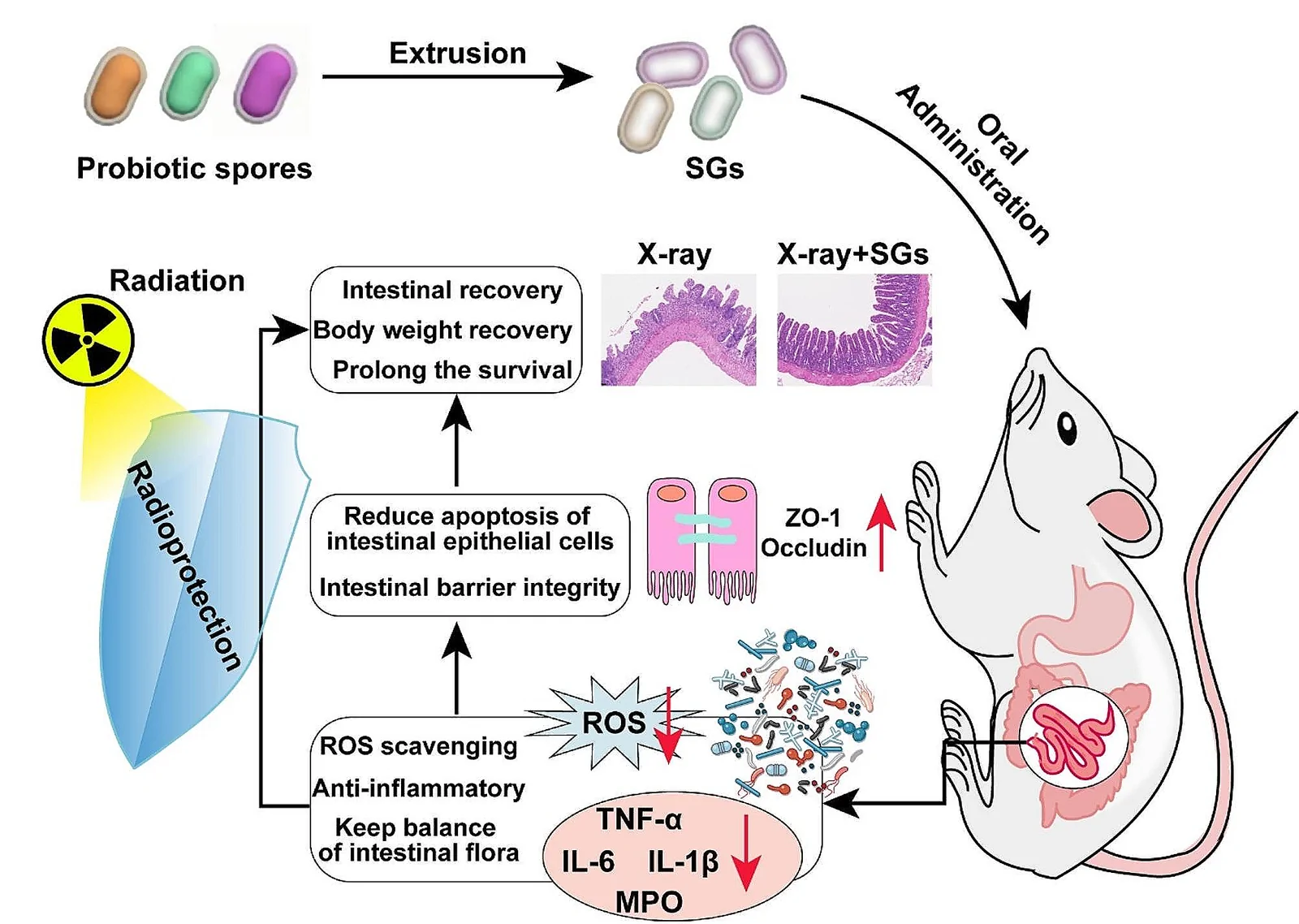
Schematic diagram of probiotic SGs on prevention of radiation-induced intestinal injury

## Results

### The preparation and characterization of SGs

**Fig. 1:**
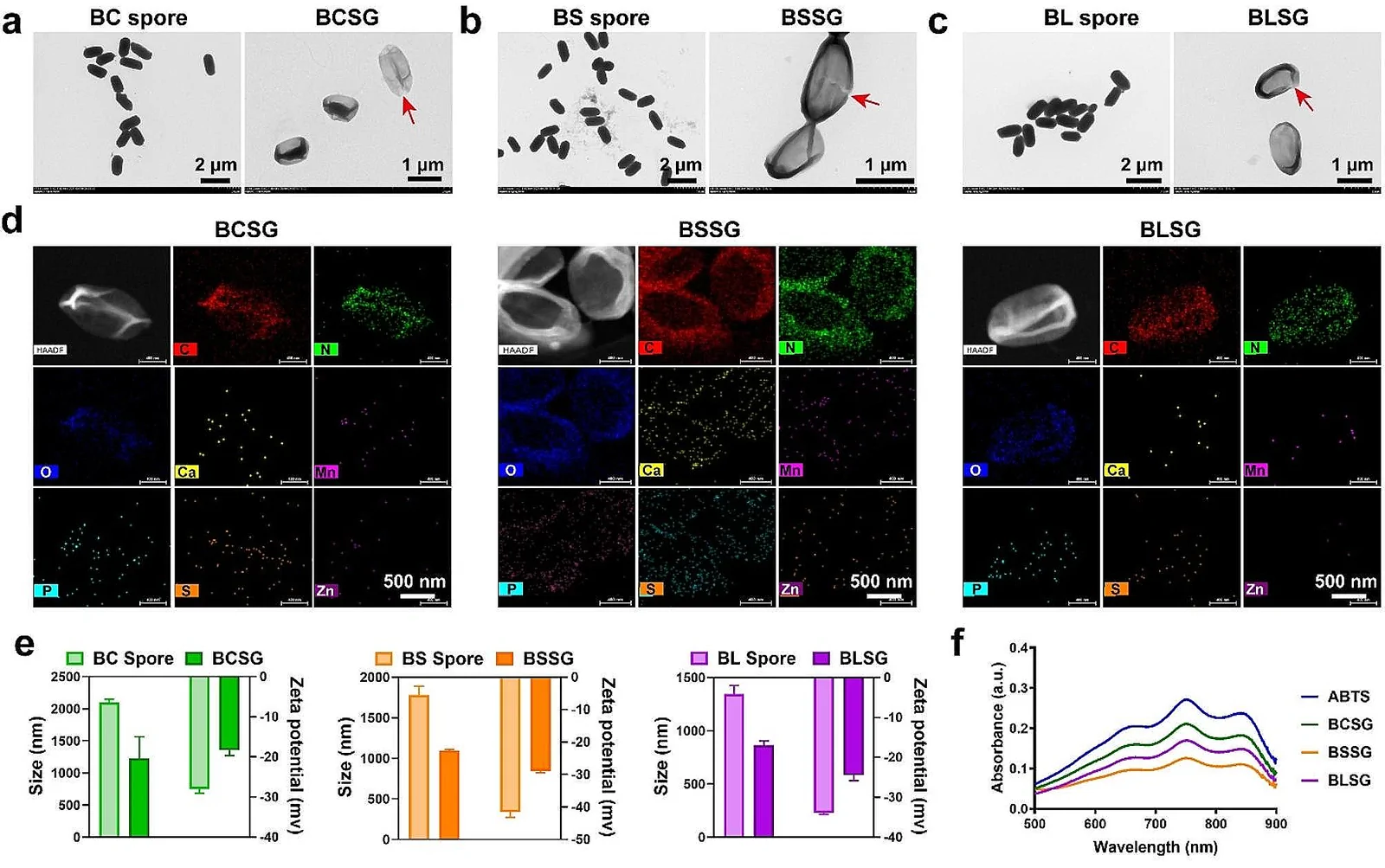
Characterization of BCSG, BSSG and BLSG. (a-c) TEM images of (**a**) BC spores and BCSG, (**b**) BS spores and BSSG, (**c**) BL spores and BLSG. (**d**) HAADF images of BCSG, BSSG and BLSG. (**e**) Size distribution and zeta potential of spores and SG (*n* = 3). (**f**) ABTS radical scavenging ability of BCSG, BSSG and BLSG.

The probiotic spores were separated from *B.coagulans, B.subtilis* and *B.licheniformis* when these probiotics were cultured in sporulation medium for 48 h. From the transmission electron microscope (TEM) images (Fig. 1a ~ c), the spores of 1 ~ 2 μm with plump and integrity shape were separated successfully. Then, SGs of *B.coagulans* (BCSG), *B.subtilis* (BSSG) *and B.licheniformis* (BLSG) were prepared following our previous literature procedure with a minor modification [34]. As shown in Fig. 1a ~ c, main contents were released from spores through the clear breaches pointed by red arrows under mechanical force, indicating that SGs were isolated successfully. Of note, the hydrodynamic size and zeta potential of SGs were slightly smaller than their parent spores (Fig. 1e and S1), which was mainly due to the morphology shrinkage after the contents release. After freeze-drying, BLSG had the highest productivity of ~ 33.28%, which was slightly higher than that of BCSG (~ 27.44%) and significantly higher than that of BSSG (~ 14.73%). Next, we analyzed the distribution of different elements on these SGs by elemental mapping. As shown in Fig. 1d, Fig. S2 and Table S1, the three SGs contained C, N, O, Ca, Mn, P, S elements. BCSG and BSSG contained amount of C, N, O elements and relatively low content of other elements. Unlikely, BLSG contained Zn ions, although the Zn content was lower than other elements. Moreover, the amounts of Ca, Mn, P, S in BLSG were significantly higher than that of BCSG and BBSG. Among these elements, C, N, O came from the domain and binding proteins of SGs. Ca ions could be related to the repair of epithelial barrier [35]. Mn ions could promote explosive proliferation of intestinal flora by providing the extra active substances [31]. Zn ions could lead to a superior inhibition of proliferation of harmful bacteria by competitive colonization [36]. 

Free radical scavenging has always been regarded as one of the most efficient methods towards radioprotection [37]. Therefore, we detected radical scavenging ability of the three SGs by using classical model radical 2,2′-azinobis (3-ethylbenzthiazoline-6-sulfonate) (ABTS), and preliminarily evaluated their radioprotection potential. As shown in Fig. S3, lower characteristic absorbance of ABTS was observed after treatment with these SGs. Although all the three SGs could scavenge ABTS free radical in a concentration-dependent manner, the scavenging ability of BSSG for ABTS was higher than those of BCSG and BLSG under the same condition (Fig. 1f). Consequently, the results demonstrated that the three SGs of probiotics with free radical scavenging ability had good potential as radioprotective agents.

### In vitro radioprotective effect

**Fig. 2:**
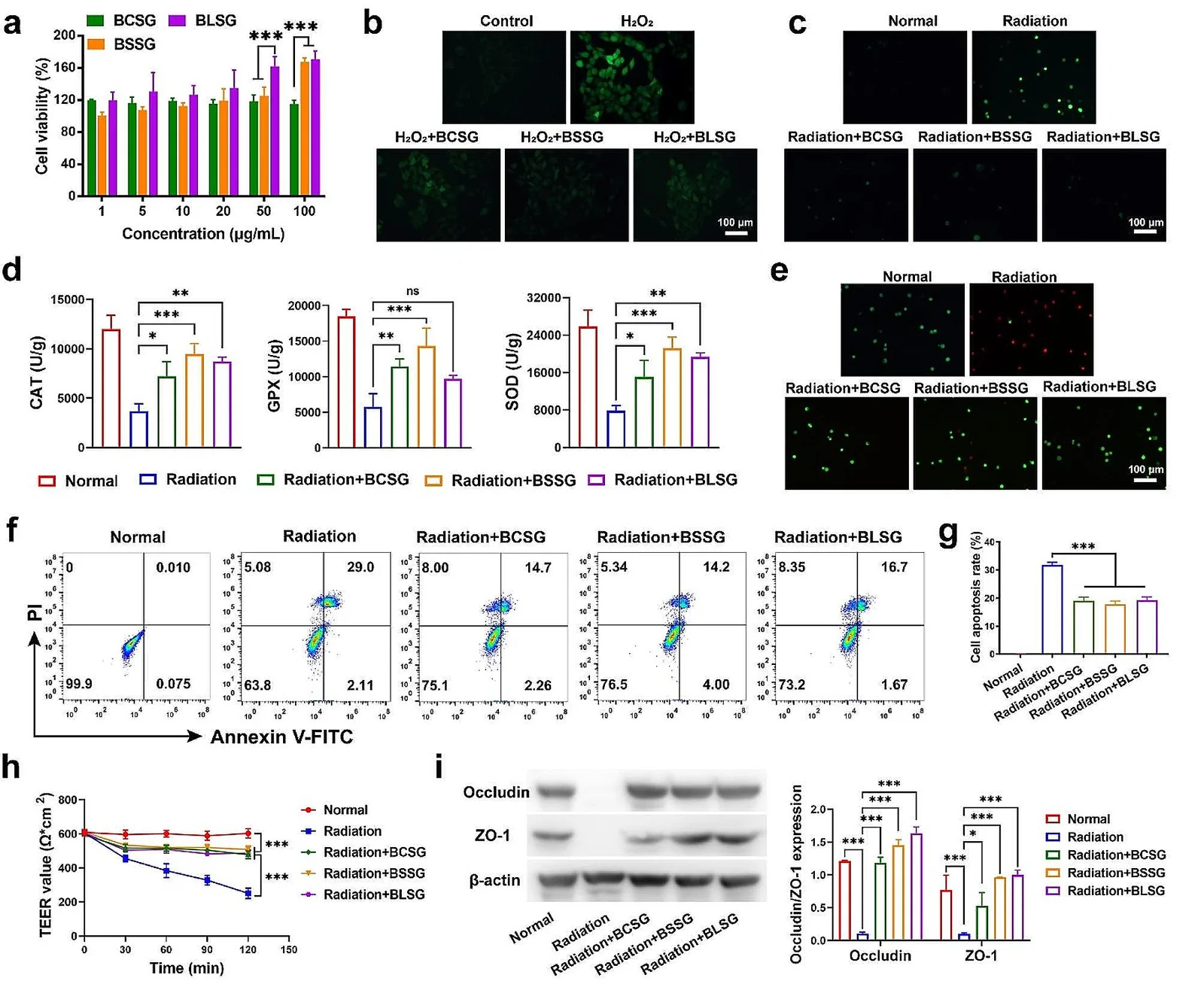
Radioprotection effects of BCSG, BSSG and BLSG in IEC-6 cell model. (**a**) Cytotoxicity assays on IEC-6 cells after treatment with different concentrations of BCSG, BSSG and BLSG. (**b**) Effect of SGs on H_2_O_2_-induced intracellular level of total ROS. (**c**) Corresponding fluorescence images of intracellular ROS. (**d**) Activities of CAT、GPX and SOD. (**e**) Calcein-AM/PI fluorescence images of the IEC-6 cells irradiated by 4.5 Gy X-ray. (**f, g**) Apoptosis level of IEC-6 cells. (**h**) TEER values of Caco-2 cell monolayers after different treatment. (**i**) Expression of occluding and ZO-1. Data are presented as mean ± SD (*n* = 3). *p* values were calculated by one-way or two-way ANOVA with a Tukey post-hoc test (*p < 0.05, **p < 0.01 and ***p < 0.001)

Inspired by the free radical scavenging ability of SGs, the radioprotective ability of these SGs was estimated in vitro by using the rat small intestinal crypt epithelial cells (IEC-6 cells). In order to explore biomedical application potential of probiotic SGs, we first employed CCK8 assay to investigate toxicity of the three SGs on IEC-6 cells. As shown in Fig. 2a, no cytotoxicity of IEC-6 cells was observed after incubation with all the three SGs for 24 h. Notably, the viability of IEC-6 cells was significantly increased after treatment with 100 µg/mL BSSG and BLSG. Moreover, the viability BLSG group increased in a concentration-dependent manner, which might be attribute to abundant elements in BLSG than BCSG and BSSG. Next, the in vitro ROS scavenging experiment was conducted in culture medium containing H_2_O_2_ to simulate irradiation induced oxidative microenvironment. When the IEC-6 cells were co-incubated with SGs, intracellular ROS level was significantly decreased, as demonstrated in Fig. 2b.

Next, a series of experiments were conducted to estimate the radioprotective ability of these probiotic SGs for IEC-6 cells. As shown in Fig. 2c, intracellular ROS level was obviously increased after treatment with 4.5 Gy X-ray radiation. However, pre-incubation with all the three SGs could significantly inhibit intracellular ROS production induced by X-ray radiation. It is common knowledge that antioxidant enzymes form the first line of defense against free radical damage to organisms [38, 39]. Therefore, we investigated the activities of antioxidative enzymes, including antioxidase catalase (CAT), glutathione peroxidase (GPX) and superoxide dismutase (SOD). As illustrated in Fig. 2d, BCSG, BSSG and BLSG could reverse X-ray radiation induced activity loss of the main antioxidative enzymes, which was an important contribution to the radioprotection effects of probiotic SGs.

Subsequently, we evaluated the injury of IEC-6 cells exposed to 4.5 Gy X-ray with the culture medium containing different probiotic SGs. Calcein-AM/PI double staining (Fig. 2e) assay showed that pre-incubation with all the three SGs could significantly improve viability of irradiated cells. The same result was also obtained from the experimental study on apoptosis of IEC-6 cells. As illustrated in Fig. 2f and g, after radiation with 4.5 Gy of X-ray, the apoptosis rate was around 31%. However, apoptosis rate of cells pre-treated with BCSG, BSSG and BLSG were around 17%, 18% and 18% respectively, which was significantly lower than that of X-ray radiation group. The above results indicated that the three probiotic SGs could not only scavenge intracellular ROS, but also increase the activities of antioxidative enzymes, thus enhancing the cell tolerability of radiation under the same dosage of X-ray exposure.

The disruption of tight junction is the main characteristic in radiation-induced intestinal injury [40]. Intrigued by above results, we examined the impact of probiotic SGs on intestinal barrier functions using human colon epithelial cancer cells (Caco-2). As reflected in Fig. 2h, the transepithelial electrical resistance (TEER) value of Caco-2 cell monolayer was decreased obviously after treatment with 4.5 Gy X-ray. By contrast, all the three probiotic SGs could prevent the early dysfunction of transepithelial electrical resistance value. Moreover, the three SGs could also prevent the loss of tight junction-associated proteins (E-cadherin and ZO-1), which play pivotal roles in gut homeostasis. The above results demonstrated that BCSG, BSSG and BLSG could suppress the damage of X-ray on intestinal epithelial cells and enhance the intestinal barrier functions, exhibiting excellent radioprotective potential.

### In vivo biodistribution and radioprotective effect

To visualize the biodistribution of these SGs, fluorophore (Cy5) was attached to SGs by chemical conjugation to obtain Cy5-BCSG, Cy5-BSSG and Cy5-BLSG, which were employed to estimate the residence time of these SGs in intestine. As shown in Fig. S4, most of free Cy5 dye entered the cecum 2 h post intragastric administration. Most free Cy5 was cleaned out of body within 8 h. While, much longer retention time was observed in Cy5-BCSG, Cy5-BSSG and Cy5-BLSG groups. Interestingly, no fluorescent signal was detected in blood and other main organs (heart, liver, spleen, lung, and kidney) except for the intestine at all-time points. The main reason for this phenomenon was speculated to be that free Cy5 was trapped and quickly cleared by mucus. While the SGs could hardly penetrate the intestinal barrier functions due to their micro scale.

**Fig. 3:**
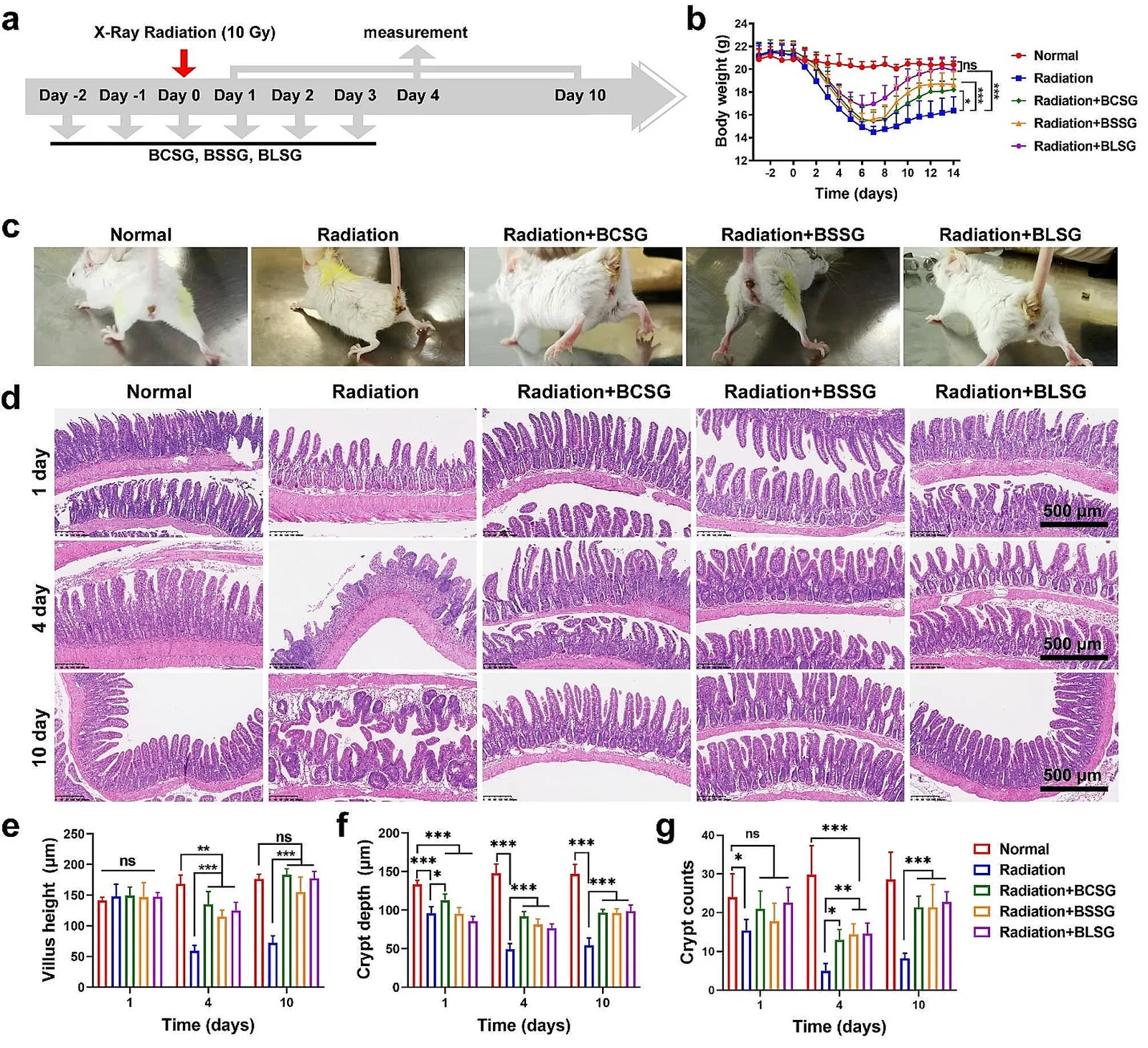
Radioprotective effects of BCSG, BSSG and BLSG in BLAB/C mouse model. (**a**) Therapeutic schedule. (**b**) The body weight changes in mice, (*n* = 15). (**c**) The images of diarrhea in mice at 4th day. (**d**) H&E staining images of intestine with different treatments after irradiation at 1st, 4th, and 10th day, (*n* = 5). (**e**) Villus height, (*n* = 5). (**f**) Crypt depth of small intestine, (*n* = 5). (**g**) Crypt counts in three visual fields of small intestine, *n* = 5. Data are presented as mean ± SD. *p* values were calculated by two-way ANOVA with a Tukey post-hoc test (*p < 0.05, **p < 0.01 and ***p < 0.001)

Next, we evaluated in vivo radioprotective effects of the three SGs according to the experimental schedule shown in Fig. 3a. Female BALB/c mice (8–10 weeks old) were randomly divided into five groups (*n* = 15): Normal, Radiation, Radiation + BCSG, Radiation + BSSG and Radiation + BLSG. To perform total abdominal radiation, anesthetized mice were placed in a lead plate in the supine position and exposed to X-ray radiation (Fig. S5). The mice treated with a sublethal dose of total abdominal X-ray radiation (TAR) were accompanied by cachexia and anorexia. Mice body weight successively decreased in the first couple of days, as displayed in Fig. 3b. The body weight of the mice was recorded every day. On the 1st, 4th, and 10th day after X-ray irradiation, five mice were sacrificed from each group to collect intestine for pathological analysis. Compared with radiation alone group, the three SGs could not only prevent the bodyweight loss in different degrees, but also promote bodyweight recovery. The three SGs treated mice started to recover bodyweight on day 7, while X-ray treated mice returned to a recovered state on day 8. On the 14th day, the average body weight showed a remarkable difference between different groups. The body weight of mice in Radiation + BLSG group almost returned to the degree of Normal group, and body weights of Radiation + BCSG and Radiation + BSSG groups were slightly lower than that of Normal group. However, the mice in Radiation alone group still weighed around 4 g less than healthy mice. Consistently, food and water intake showed the same trend as body weight change, as depicted in Fig. S6 and S7. Meanwhile, the three probiotic SGs could also alleviate X-ray induced serious diarrhea (Fig. 3c).

Subsequently, to directly verify the intestinal radioprotective effect of these SGs, intestinal tissues of mice were collected for pathological analysis at different time point. As illustrated in Fig. 3d, the intestinal tissue in Radiation alone group began to show slight impairment on the first day after X-ray radiation. While on the 4th day after radiation, intestinal tissue presented severe damage with necrosis of a large amount of epithelial cells, severe damage of goblet cell and loss of the crypt structure. Although the severe injury showed little recovery on the 10th day, the crypt–villus architecture of small intestine in mice of Radiation group still displayed incomplete structure. In contrast, the three SGs could greatly alleviated X-ray induced intestinal injury from the 1th day to the 10th day. The crypts of mice remained closely attached to the muscularis mucosa and intestinal villi presented relatively normal appearance with occasional goblet cells. Consistent with above pathological analysis, villi height, crypt depth and crypt count exhibited no significant difference among Normal, Radiation and SGs-treated groups on the 1th day after radiation. X-ray radiation significantly reduced regenerating crypts and shortened intestinal villi on the 4th day (Fig. 3e and g). Although these parameters of mice treated with probiotic SGs were still slightly decreased compared with Normal group, they obviously higher that of radiation alone group on the 4th day. Encouragingly, the villi height in SGs treated groups became comparable to Normal group, but crypts were not fully recovered on the 10th day after radiation. All these results demonstrated that the three SGs could effectively alleviate intestinal damage and exhibited powerful radioprotection ability.

**Fig. 4:**
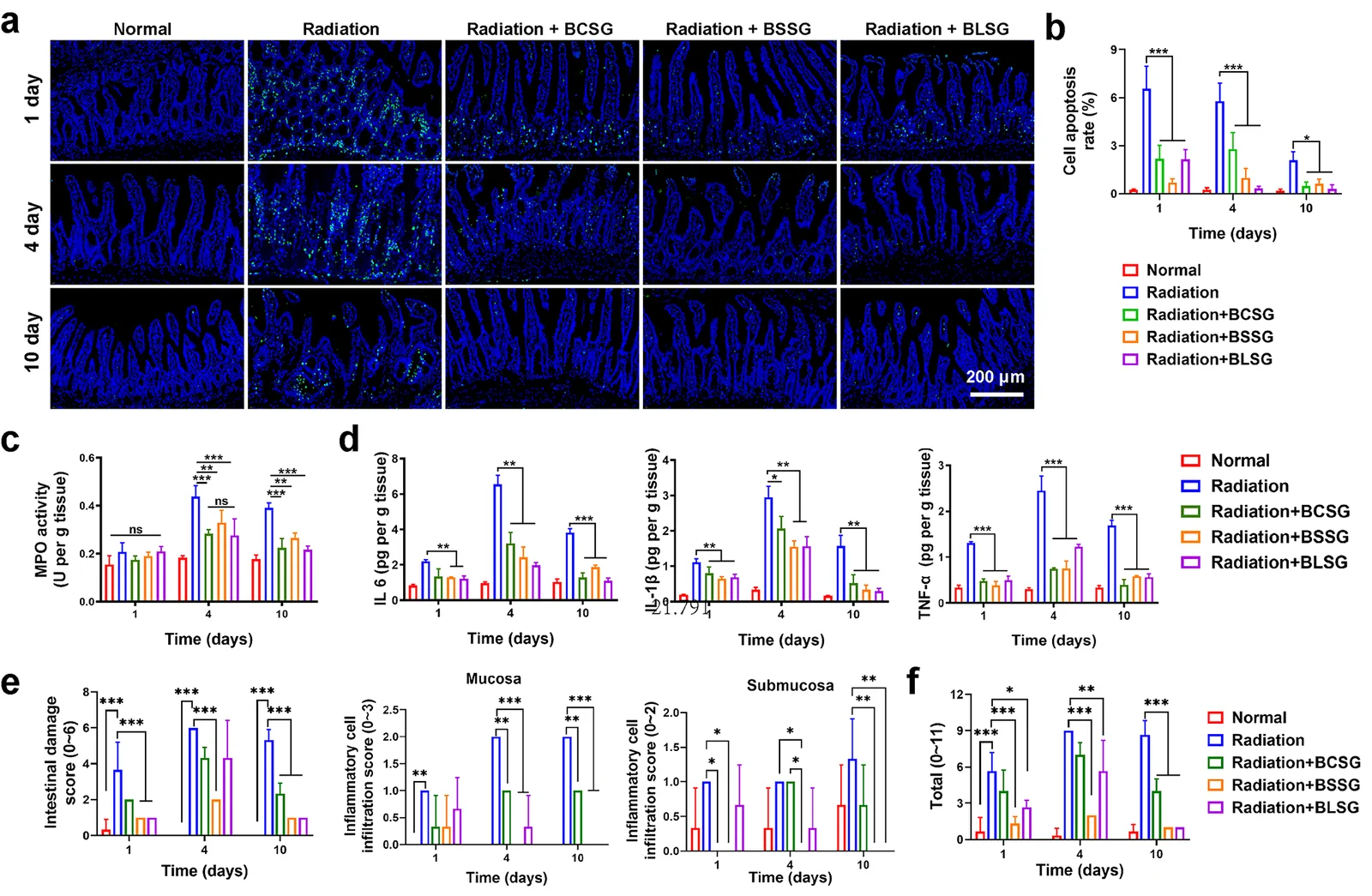
Evaluation of radioprotective effect in vivo. (**a**) Apoptosis detection of intestine sections by immunofluorescence. (**b**) The apoptosis rate of intestine cells. (**c**) Detection of MPO activity in intestine at different time points. (**d**) Detection of pro-inflammatory cytokines IL 6, IL-1β and TNF-α. (**e**) Score of intestinal damage, mucositis cell infiltration and inflammatory cell infiltration. (**f**) Total damage score of intestinal damage. Data are presented as mean ± SD (*n* = 5). *p* values were calculated by two-way ANOVA with a Tukey post-hoc test (*p < 0.05, **p < 0.01 and ***p < 0.001)

Furthermore, all the three probiotic SGs treatment markedly reduced X-ray induced apoptosis of intestinal epithelial cells, as shown by the terminal deoxynucleotidyl transferase dUTP nick end labelling (TUNEL) result in Fig. 4a and b. It is well known that radiation-induced intestinal injury is always accompanied with inflammatory response [41]. Therefore, we evaluated the activity of myeloperoxidase (MPO) and the level of pro-inflammatory cytokines in intestinal tissue after different treatments. As depicted in Fig. 4c, the increased intestinal MPO activity was significantly suppressed and down-regulated by these probiotic SGs. Meanwhile, BCSG, BSSG and BLSG treatment also significantly decreased the local levels of pro-inflammatory cytokines, including IL 6, TNF-α and IL-1β (Fig. 4d). Moreover, the decreased intestinal damage score (Fig. 4e and f), as a consequence of mucosal inflammation, further confirmed excellent anti-inflammatory potential of these SGs. In addition, we assessed the impact of X-ray radiation and probiotic SGs on intestinal barrier functions by detecting tight junction-associated protein ZO-1 and occluding on 10th day after radiation. As shown in Fig. S8 and S9, the expression of ZO-1 and occludin in Radiation group decreased obviously and significantly lower than that in BCSG, BSSG and BLSG groups, which might be attributed to Ca ions contained in these SGs. Moreover, the three probiotic SGs significantly improved weight gain (Fig. S10b) and survival (Fig. S10c) in mice after a lethal dose of TAR. The above results indicated that the three probiotic SGs had excellent ability in anti-inflammatory and enhancing of intestinal barrier functions.

**Fig. 5:**
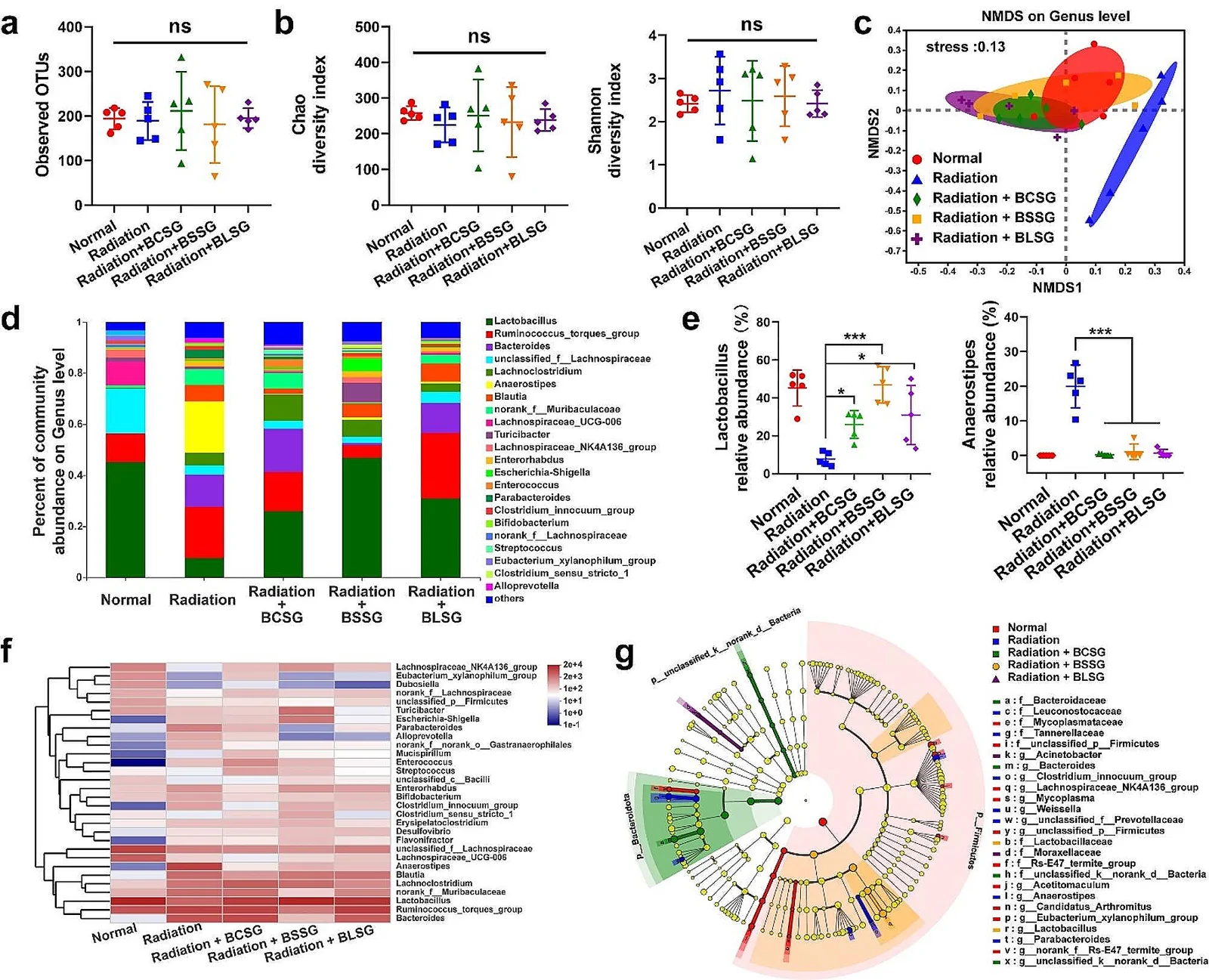
16 S rDNA gene sequencing analysis. (**a**) Estimation of microbial community observed OTU richness. (**b**) Alpha diversity boxplot, Chao and Shannon. (**c**) Beta diversity. (**d**) Relative abundance of gut microbiome. Genus-level taxonomy was presented as a percentage of total sequences. (**e**) Relative abundance of intestinal probiotics Lactobacillus and anaerobic bacteria Anaerostipes in the intestine. (**f**) Heatmap of the relative abundance of family-level taxa for each mouse. (**g**) LEfSe taxonomic cladogram, depicting the taxonomic association between the microbiome communities from different treatments. Data are presented as mean ± SD (*n* = 5). *p* values were calculated by one-way ANOVA with a Tukey post-hoc test (*p < 0.05, **p < 0.01 and ***p < 0.001)

Apart from barrier damage and inflammation response, intestinal flora imbalance has been regarded as a main reason for radiation induced intestinal injury [42, 43]. Thus, we examined the composition and abundance of gut microbiota by 16 S ribosomal DNA (16 S rDNA) identification amplicon sequencing after different treatments. Operational taxonomic units (OTUs) were clustered at 97% similarity (Fig. S11). Curiously, X-ray radiation and the three probiotic SGs had almost no impact on bacterial richness (observed OTUs richness in Fig. 5a) and diversity (Chao and Shannon in Fig. 5b). However, the non-metric multidimensional scaling (NMDS) plots revealed that mice treated with X-ray alone had distinct gut microbiota profile, compared with probiotic SGs treated groups which was the nearest to that in Normal group (Fig. 4c). Further analysis at the genus level (Fig. 4d ~ 4f) showed that X-ray treatment significantly decreased the relative abundance of *Lactobacillus* (known for beneficial roles in radioprotection) [24, 25], which was dramatically reversed by pretreatment of BCSG, BSSG and BLSG. On the contrary, the abundance of the pathogenic bacteria of Anaerostipes showed a great increase after X-ray radiation, which was considerably higher than that in Normal group and the probiotic SGs treated groups. Furthermore, the LEfSe analysis demonstrated that the probiotic SGs, especially BSSG, exhibited an efficient protective ability for *Lactobacillus*, so as to promote its large proliferation to become the dominating flora. Above results indicated that the three SGs took orally by mice could maintain the balance of intestinal flora, which was beneficial for alleviation radiation enteritis.

In addition to radiation enteritis, radiation can also cause damage to other tissue, such as hematopoietic system and immune system [44, 45]. Next, we primarily evaluated whether probiotic SGs treatment showed protective effect on other radiation related injuries. As indicated in Fig. S12, X-ray radiation caused sharp weight loss of spleen (The largest blood storage and immune tissue). However, pretreatment of BCSG, BSSG and BLSG could not reverse X-ray induced spleen damage. Similarly, these probiotic SGs were powerless in preventing X-ray induced decrease of white blood cells (WBC) and platelet (PLT), as reflected in Fig. S13. These results demonstrated that the three probiotic SGs exhibited no radioprotective effect on radiation induced damage to hematopoietic system and immune system.

**Fig. 6:**
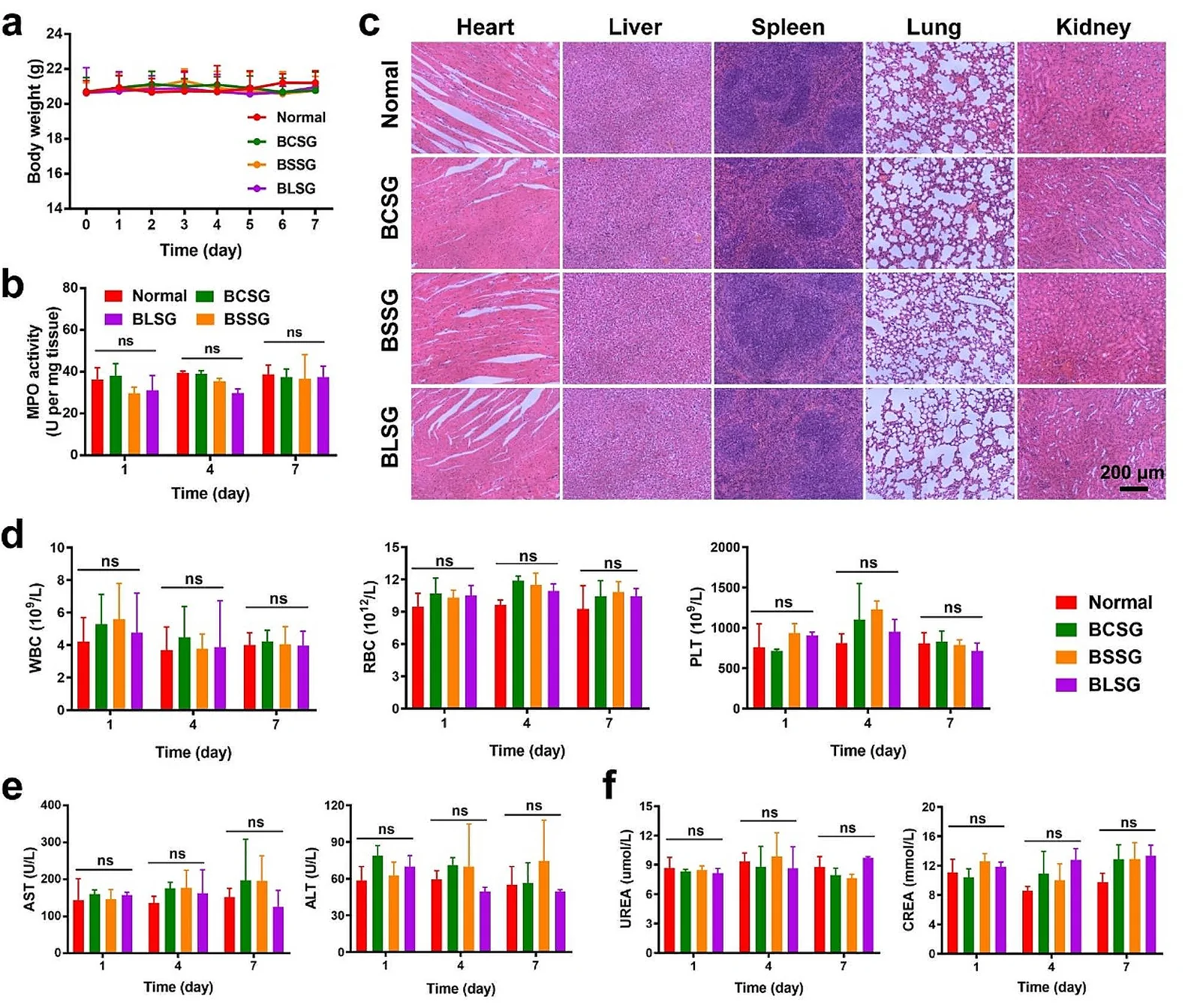
The biosafety of BCSG, BSSG and BLSG in vivo. (**a**) The body weight changes in mice. (**b**) Detection of MPO activity in intestine. (**c**) The histopathology analysis of main tissues after different treatments. (**d**) The blood routine examination at different time point. (**e, f**) The blood biochemistry analysis of (**e**) liver function and (**f**) kidney function. Data are presented as mean ± SD (*n* = 3). *p* values were calculated by two-way ANOVA with a Tukey post-hoc test

### Investigation of biosafety

Biosafety is the top priority for biomedical application and clinical transformation of radioprotectants [46]. *B.coagulans, B.subtilis* and *B.licheniformis* are CFDA-approved probiotics for the treatment of gastrointestinal disorder, so the biosafety of their spore had been proved in our previous research [33]. In this study, the three probiotic SGs were also verified to show no distribution and retention in other vital organs except for intestine via oral administration (Fig. S4), preliminarily indicating their good biosafety. Herein, the biosafety and biocompatibility of the three probiotic SGs were further evaluated by body weight, intestinal MPO, histopathology examination, blood routine, and blood biochemistry. As shown in Fig. 6a, there was no body weight loss in mice treated with BCSG, BSSG and BLSG during the test time. After oral administration, intestinal MPO in all mice treated with the three probiotic SGs showed no significant difference with healthy mice (Fig. 6b). In addition, no noticeable damages of the main organs (heart, liver, spleen, lung, and kidney) were detected, according to the images of hematoxylin and eosin (H&E) staining (Fig. 6c). Moreover, the blood hematology (Fig. 6d), liver function (Fig. 6e) and renal function (Fig. 6f) exhibited no significant changes after oral administration with the three probiotic SGs. Consequently, these results further demonstrated the biosafety of BCSG, BSSG and BLSG.

## Conclusion

In conclusion, three different probiotic SGs separated from the spores of *B.coagulans, B.subtilis and B.licheniformis* were explored for their radioprotective effect in vitro and in vivo. We found that all the three SGs could scavenge ABTS free radical in a concentration-dependent manner, especially BSSG, which was further confirmed by in vitro ROS scavenging experiment. Moreover, the regulation of the three SGs on main antioxidative enzymes and ROS scavenge synergistically prevented the X-ray induced damage on IEC-6 cells. Furthermore, we performed total abdominal X-ray radiation on mice using a sublethal dose of the therapeutic effects and a lethal dose for survival analysis. These probiotic SGs demonstrated excellent therapeutic effects on radiation-induced intestinal injury by relieving diarrhea, preventing crypt and villus destruction, inhibiting inflammation, enhancing intestinal barrier function and maintaining the stability of gut microbiota. Therefore, all the three SGs obviously prevented bodyweight loss and promoted bodyweight gain after X-ray radiation, among which mice in BLSG group even achieved full bodyweight recovery. Importantly, all the three SGs improved the survival rate of mice after lethal dose of total abdominal radiation. Consequently, the BCSG, BSSG and BLSG exhibited well potential as intestinal radioprotectants for radiation induced intestinal injury. Moreover, in-depth examinations that combine metabolomics are required in future studies. Accordingly, based on excellent radioprotective effect and good biosafety in vivo, we are looking forward to their further clinical translation for intestinal radioprotection.

## Electronic supplementary material

Below is the link to the electronic supplementary material.

Supplementary Material 1

## Data Availability

No datasets were generated or analysed during the current study.
