# Correction: Oral administration of probiotic spore ghosts for efficient attenuation of radiation-induced intestinal injury

**DOI:** 10.1186/s12951-026-05129-z

**Published:** 2026-10-13

**Authors:** Cuixia Zheng, Mengya Niu, Yueyue Kong, Xinxin Liu, Junxiu Li, Xunwei Gong, Xinyuan Ren, Chen Hong, Menghao Yin, Lei Wang

**Affiliations:** 1 School of Pharmaceutical Sciences, Zhengzhou University, Zhengzhou, 450001 China; 2 Translational Medicine Center, Huaihe Hospital of Henan University, Kaifeng, 475000 China; 3 Pingyuan Lab, Henan Normal University, Xinxiang, 453007 China; 4 Xinjiang Aksu First People’s Hospital, Akesu, 843000 China; 5 Luoyang Central Hospital Affiliated to Zhengzhou University, Luoyang, 471009 China

**Correction: Journal of Nanobiotechnology (2024) 22:303**

**https://doi.org/10.1186/s12951-024-02572-8**

In this article, Figure 4a appeared incorrect. The representative immunofluorescence image in radiation group of day 4 was erroneously represented by an image from radiation group of day 1. This error occurred during the figure assembly process. For completeness and transparency, the old incorrect and correct versions of Fig. 4 are displayed below.

**Incorrect Fig. 4**

**Fig. 4:**
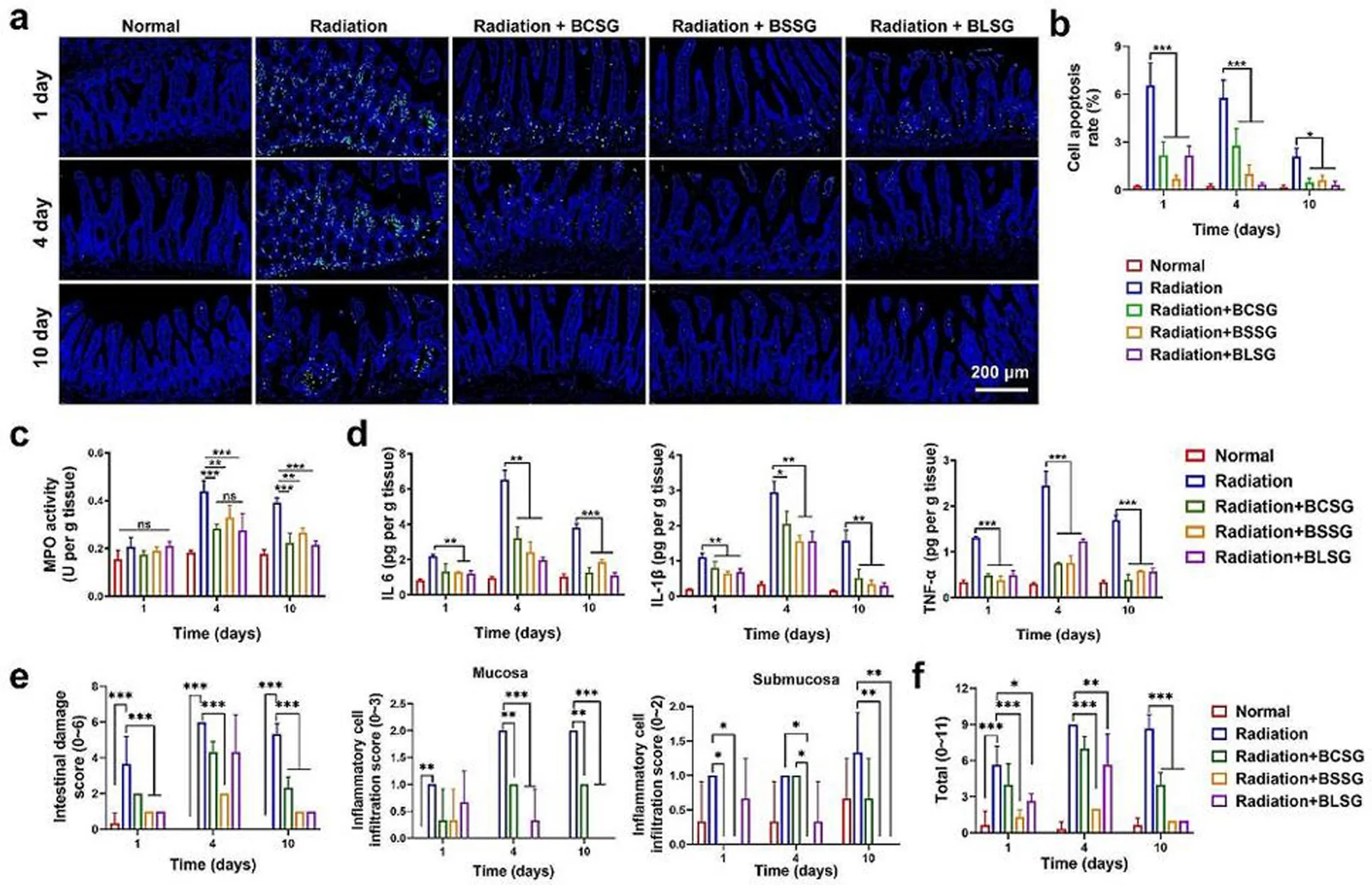
Evaluation of radioprotective effect in vivo. (**a**) Apoptosis detection of intestine sections by immunofluorescence. (**b**) The apoptosis rate of intestine cells. (**c**) Detection of MPO activity in intestine at different time points. (**d**) Detection of pro-inflammatory cytokines IL 6, IL-1β and TNF-α. (**e**) Score of intestinal damage, mucositis cell infiltration and inflammatory cell infiltration. (**f**) Total damage score of intestinal damage. Data are presented as mean ± SD (n = 5). *p* values were calculated by two-way ANOVA with a Tukey post-hoc test (*p < 0.05, **p < 0.01 and ***p < 0.001)

**Correct Fig. 4**

**Fig. 4:**
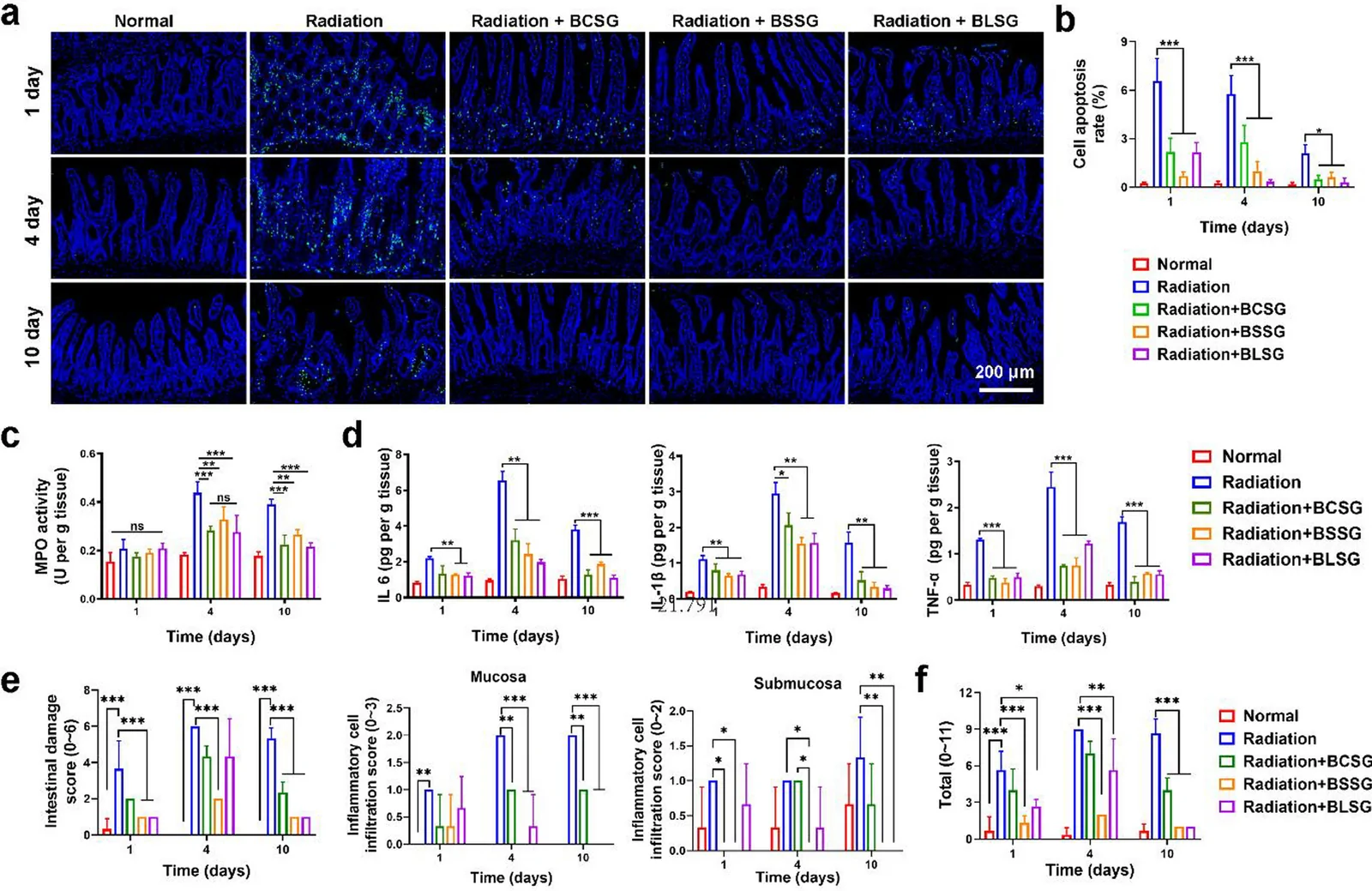
Evaluation of radioprotective effect in vivo. (**a**) Apoptosis detection of intestine sections by immunofluorescence. (**b**) The apoptosis rate of intestine cells. (**c**) Detection of MPO activity in intestine at different time points. (**d**) Detection of pro-inflammatory cytokines IL 6, IL-1β and TNF-α. (**e**) Score of intestinal damage, mucositis cell infiltration and inflammatory cell infiltration. (**f**) Total damage score of intestinal damage. Data are presented as mean ± SD (n = 5). *p* values were calculated by two-way ANOVA with a Tukey post-hoc test (*p < 0.05, **p < 0.01 and ***p < 0.001)

The original article has been corrected.

