# Supplementary material for: Oral administration of probiotic spore ghosts for efficient attenuation of radiation-induced intestinal injury
Source: J Nanobiotechnology. 2024 May 31;22:303. doi: 10.1186/s12951-024-02572-8 (PMC11140926; doi:10.1186/s12951-024-02572-8)
Supplement: Supplementary file 1 — Supplementary Material 1 [file 12951_2024_2572_MOESM1_ESM.docx]

**Oral Administration of Probiotic Spore Ghosts for Efficient** **Attenuation of** **Radiation-Induced Intestinal Injury**

Cuixia Zheng^2^, Mengya Niu^1^, Yueyue Kong^4^, Xinxin Liu^1,5^, Junxiu Li^1^, Xunwei Gong^1^, Xinyuan Ren^1^, Chen Hong^2^, Menghao Yin^1^, Lei Wang^1,3,5*^

^1^School of Pharmaceutical Sciences, Zhengzhou University, Zhengzhou 450001, China

^2^Huaihe Hospital of Henan University, Translational medicine Center, Kaifeng 475000, China

^3^Pingyuan Lab, Henan Normal University, Xinxiang 453007, China

^4^Xinjiang Aksu First People's Hospital, Akesu 843000, China

^5^Luoyang Central Hospital Affiliated to Zhengzhou University, Luoyang 471009, China

Tel.: +86-371-67781910, E-mail: wanglei1@zzu.edu.cn (L. Wang).

**Materials and methods**

**Materials**. Bacillus coagulans (BC), Bacillus subtilis (BS) and Bacillus licheniformis (BL) were obtained from BeNa Chuang Lian Biotechnology Research Institute (Beijing, China). Myeloperoxidase (MPO) assay kit was obtained from NanJing JianCheng Bioengineering Institute Co., Ltd. IL 6 Elisa kit, IL-1β Elisa kit and TNF-α Elisa kit were both purchased from MULTI SCIENCES (Hangzhou, China).

**The preparation and characterization of spore ghosts (SG)**. Firstly, the culture of BC, BS and BL and separation of their spores were referred to the methods that we have previously reported. The morphologies of probiotic spores were characterized by 150kV Transmission Electron Microscope (TEM, Hitachi, HT7700, Japan). For the preparation of SG, the spore solution was diluted to 0.5 mg/mL and then extruded back and forth for 13 times through polycarbonate membrane with pore size of 800 nm using a micro extruder (LiposoEasy LE-1, MORGEC, America). Next, the mixture solution was centrifuged at 5000 rpm for 10 min and followed by washing with deionized water for 3 times to obtain the SG. The morphology, size distribution and zeta potential of CN were evaluated by the TEM and Zetasizer Nano ZS-90 instrument (Malvern, UK), respectively. And the high-angle annular dark-field scanning TEM based elemental mapping further confirmed the distribution of multiple elements on CN.

**ABTS free radical scavenging assay**. 7 × 10^−3^ M ABTS solution was reacted with the 2.45 × 10^−3^ m potassium persulfate in the dark for 16 h to obtain the ABTS radical. Then, anhydrous ethanol was utilized to dilute the ABTS radical solution until the absorbance value at 734 nm was about 0.7 to attain the ABTS radical working solution. Subsequently, a series of the BCSG, BSSG and NLSG solution were prepared with different concentrations (1, 2, 5, 10, 25, 50 and 100 µg/mL). Next, the ABTS working solution was mixed with the equal volume of BCSG, BSSG and NLSG solution, respectively. The mixture was completely reacted in the dark for 10 min. Finally, the absorbance value at 734 nm of each group was monitored by ultraviolet-visible (UV-vis) absorption spectroscopy.

**Cells viability assay detection**. IEC-6 cells were cultured in 96-well plates at the density of 8 × 10^3^ cells per well with the complete medium. 24 h later, the cells were treated with a series of concentrations of BCSG, BSSG and BLSG. After 24 h treatment, the cell viability was detected by thiazolyl blue (MTT) experiment.

**Intracellular ROS assay**. IEC-6 cells were cultured in 6-well plates at the density of 3 × 10^5^ cells per well with the complete medium. After attachment, the cells were incubated with BCSG, BSSG and BSSG at the concentration of 50 μg/mL. After 4 hours, the cells were treated with X-ray irradiation at the dosage of 4.5 Gy. After incubation for another 24 h, the cells were incubated with 2,7-Dichlorodihydrofluorescein diacetate (DCFH-DA) probes. Then, cells were washed with phosphate buffered saline (PBS) and captured by the laser microscope.

**Live and Dead cell assay**. IEC-6 cells were cultured in 6-well plates at the density of 3 × 10^5^ cells per well with the complete medium. 24 h later, the cells were incubated with BCSG, BSSG and BSSG at the concentration of 50 μg/mL. After 4 hours, the cells were treated with X-ray irradiation at the dosage of 4.5 Gy. After incubation for another 24 h, the cells were stained by Calcein-AM/PI double stain kit and observed by a fluorescence microscope.

**Cell apoptosis detection**. IEC-6 cells were cultured in 6-well plates at the density of 3 × 10^5^ cells per well with the complete medium. 24 h later, the cells were incubated with BCSG, BSSG and BSSG at the concentration of 50 μg/mL. After 4 hours, the cells were treated with X-ray irradiation at the dosage of 4.5 Gy. After incubation for another 24 h, Annexin V-FITC/PI detection kit was used to detect cell apoptosis.

**Animals**. All the work performed on animals was in accordance with the Guidelines for Care and Use of Laboratory Animals of Zhengzhou University, and the experiments were approved by the Animal Ethics Committee of Zhengzhou University. All the animals were obtained from Beijing SPF Biotechnology Co., Ltd.

***In vivo* biodistribution of BCSG, BSSG and BLSG**. Firstly, fluorophore (Cy5) was attached SG to by chemical conjugation amino on the surface of BCSG, BSSG and BLSG to the carboxylic acid of Cy5-COOH. Next, female BALB/c mice were selected to testify the in vivo biodistribution of SG after oral administration at different times. Mice were oral administrated with Cy5, Cy5-BCSG, Cy5-BSSG and Cy5-BLSG at the same dosage of Cy5. At different time point, the mice were sacrificed. Fluorescence intensities in main organs and blood were analyzed using a *in vivo* imaging system (Bruker).

**Radiation protection experiment *in vivo***. Eight-ten-week-old female BALB/c mice were randomly divided into five groups (n=15) including Normal, Radiation, Radiation + BCSG, Radiation + BSSG and Radiation + BLSG. Among these groups, the mice in normal and Radiation groups were treated with saline. And the mice in Radiation + BCSG, Radiation + BSSG and Radiation + BLSG groups were orally administered 0.5 mg/kg corresponding SG dispersed in 100 μL of saline for successive 6 days. To perform total abdominal radiation anesthetized mice were placed in a lead plate in the supine position. The abdomen (up to diaphragm, down to pelvis) of mice in radiation groups were exposed to 10 Gy of X-ray at the dose rate of 1 Gy/min (PXI-X RAD320 irradiator). The body weights, food intake and water consumption were measured every day. After being irradiated for 1, 4, and 10 days, small intestine, spleen and blood of mice in each group were collected to evaluate the effect of radioprotection of SG.

**Histology detection**. For histological analyses, small intestines collected from sacrificed mice were fixed with formaldehyde (4%). Then, the small intestines (Ileum segment) were stained with hematoxylin and eosin (H&E) for further pathological analysis. Firstly, villus height, crypt depth and crypt count were counted. Secondly, TdT-mediated dUTP nick end labeling (TUNEL) assay was used to detected cell apoptosis of small intestines. Then, Intestinal damage was assigned scores as follows: 0, normal; 1, hyperproliferation, irregular crypts, and goblet cell loss; 2, mild to moderate crypt loss (10–50%); 3, severe crypt loss (50–90%); 4, complete crypt loss, surface epithelium intact; 5, small-to-medium-sized ulcers (<10 crypt widths); 6, large ulcers (≥10 crypt widths). Inflammatory cell infiltration was scored separately for the mucosa (0, normal; 1, mild; 2, modest; 3, severe) and submucosa (0, normal; 1, mild to modest; 2, severe).

**MPO activity measurement**. Firstly, an intestine segment (1:10 w/v) in cold PBS was homogenized in a KZ-II high speed tissue grinder (Servicebio, China) at 4 °C. The supernatant of tissue homogenate was collected after centrifugation at 12000 g for 10 min. MPO activity in the intestine tissues was detected by using an MPO determination kit.

***In vivo* enzyme-linked immunosorbent assay (ELISA) analysis**. Firstly, an intestine segment (1:10 w/v) in cold PBS was homogenized in a KZ-II high speed tissue grinder (Servicebio, China) at 4 °C. The supernatant of tissue homogenate was collected after centrifugation at 12000 g for 10 min at 4 °C. The levels of IL 6, IL-1β and TNF-α in the resulting supernatants were measured by ELISA according to the instructions.

**Detection of intestinal microorganisms**. At the end of experiment, the cecum contents of mice were collected and stored in at −80 °C refrigerator. The sequencing of intestinal flora was charged with Shanghai Major Biotechnology Co., Ltd. Subsequently, the 16S rDNA amplicon sequencing method was used for the determination of the flora. Briefly, the genomic DNA was extracted, tested, and carried on PCR amplification, respectively. And then the extracted DNA was used to generate 16S rDNA libraries for community analysis using the NovaSeq sequencing platform.

**Biological safety evaluation**. Female BALB/c mice were divided into four groups: Normal, BCSG, BSSG and BLSG. The mice in normal group were treated with saline. And the mice in BCSG, BSSG and BLSG groups were orally administered 0.5 mg/kg corresponding SG dispersed in 100 μL of saline. After 1, 4 and 7 days, 3 mice in each group were sacrificed. The blood was collected for blood hematology and biochemistry detection, and the intestinal tissues were gathered for MPO detection, and the major organs were gathered for histopathology analyses.

**Statistical analysis**. The data were presented as mean ± standard deviation (SD). A one-way or two-way analysis of variance (ANOVA), followed by Tukey’s honestly significant difference (HSD) multiple comparison post hoc test was used for testing differences among groups. Statistical significance was indicated as *p < 0.05, **p < 0.01 and ***p < 0.001. GraphPad Software was used for statistical analyses.


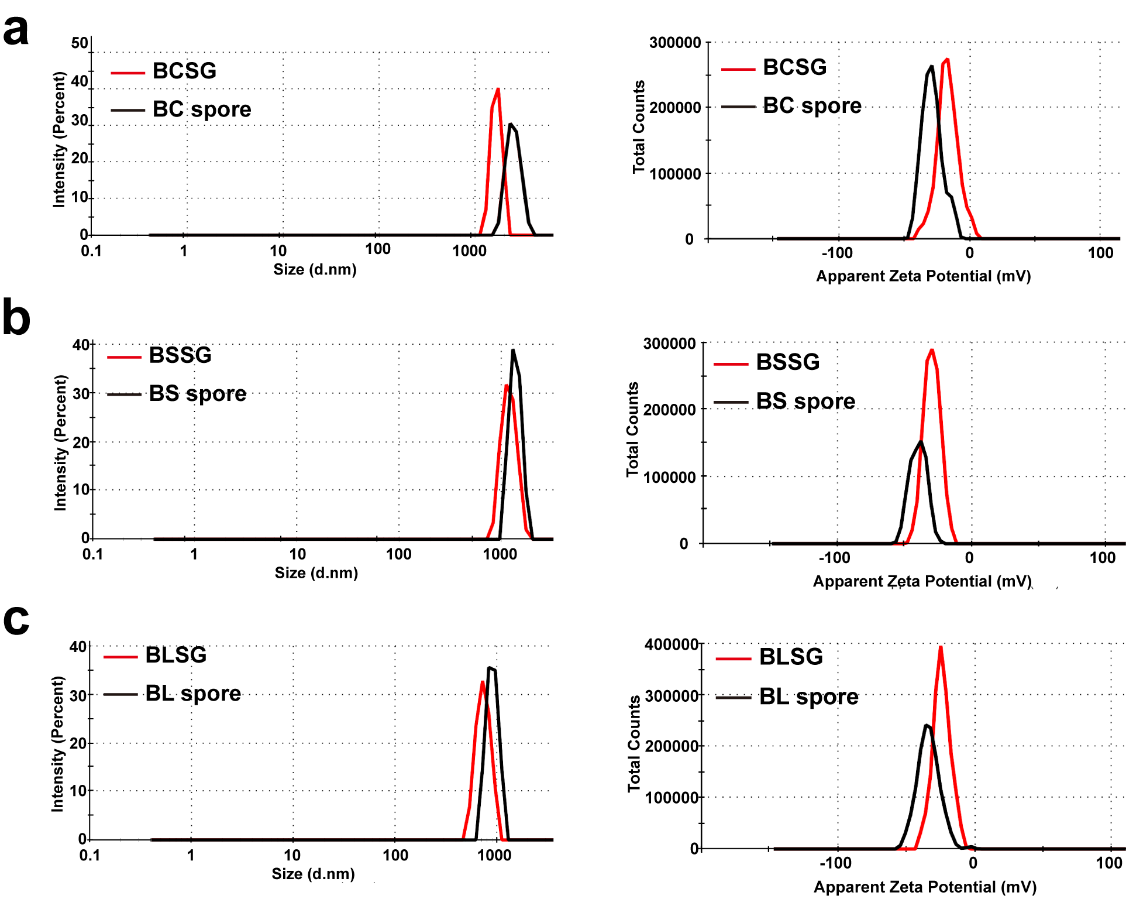


Figure S1. Size distribution and zeta potential of (a) BC spore and BCSG, (b) BS spore and BSSG, (c) BL spore and BLSG.


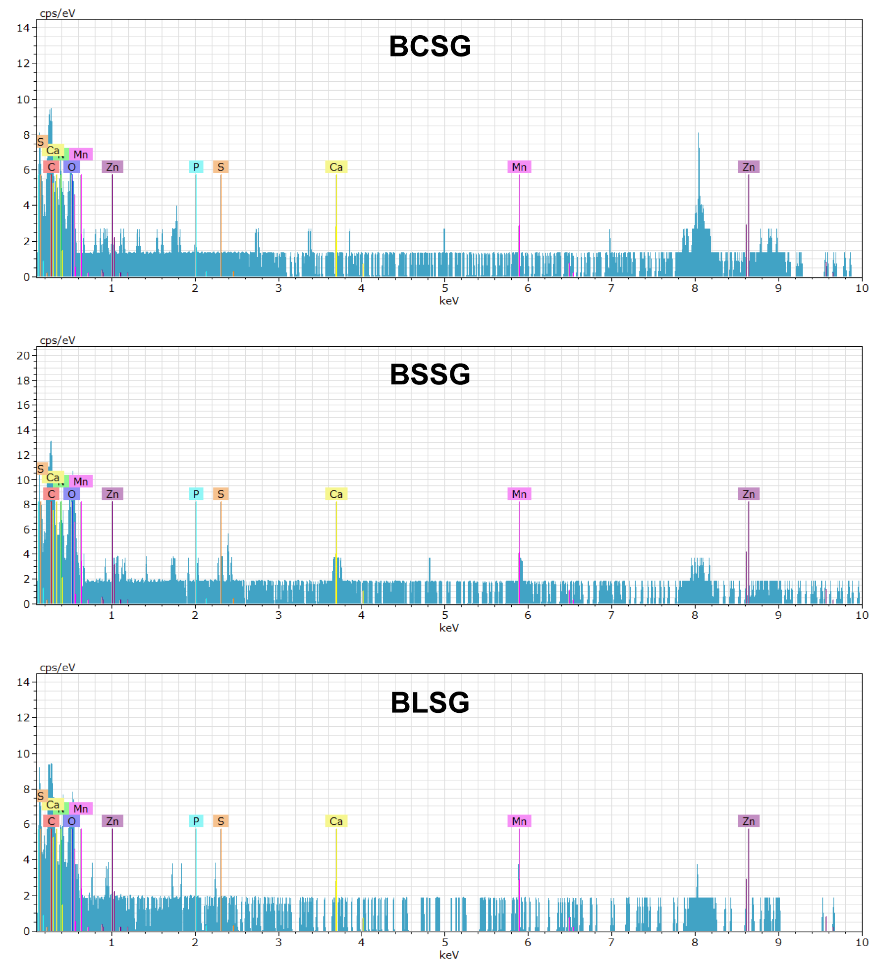


Figure S2. EDS spectrum of BCSG, BSSG and BLSG.


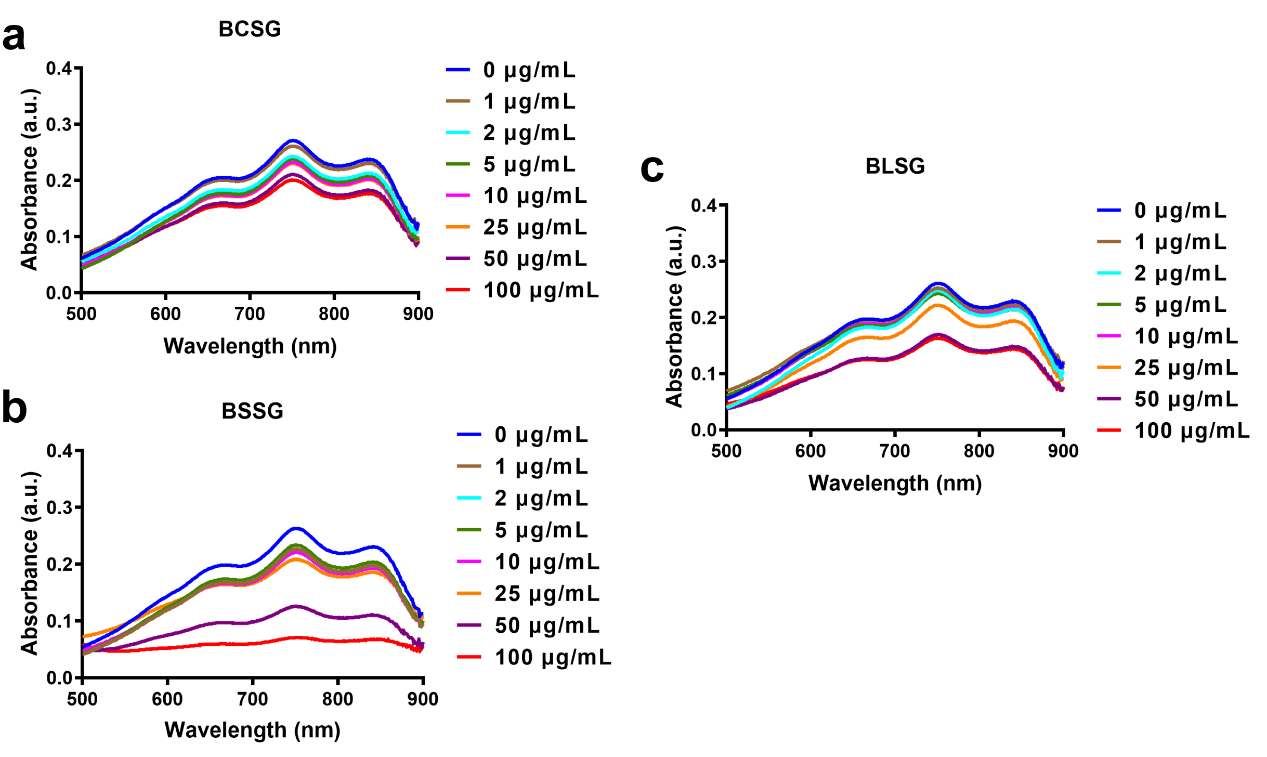


Figure S3. ABTS radical scavenging ability of BCSG, BSSG and BLSG at different concentrations.


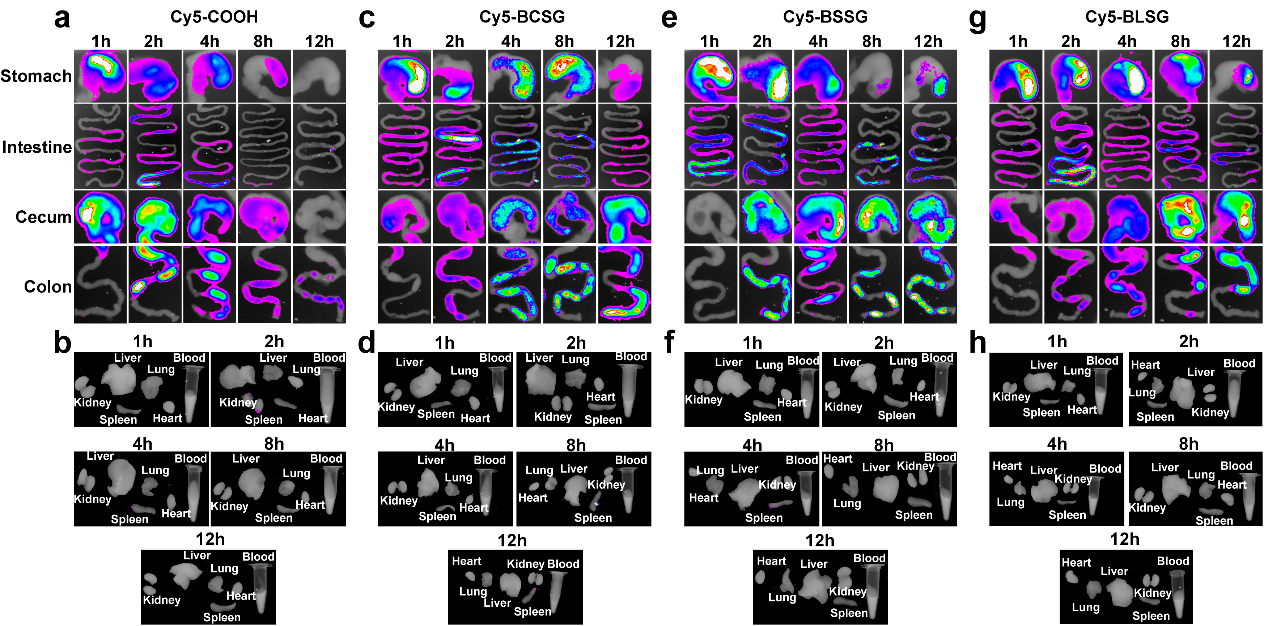


Figure S4. Near infrared fluorescence imaging at different time points. (a) Stomach, small intestine, cecum and colon. (b) Heart, liver, spleen, lung and kidney.


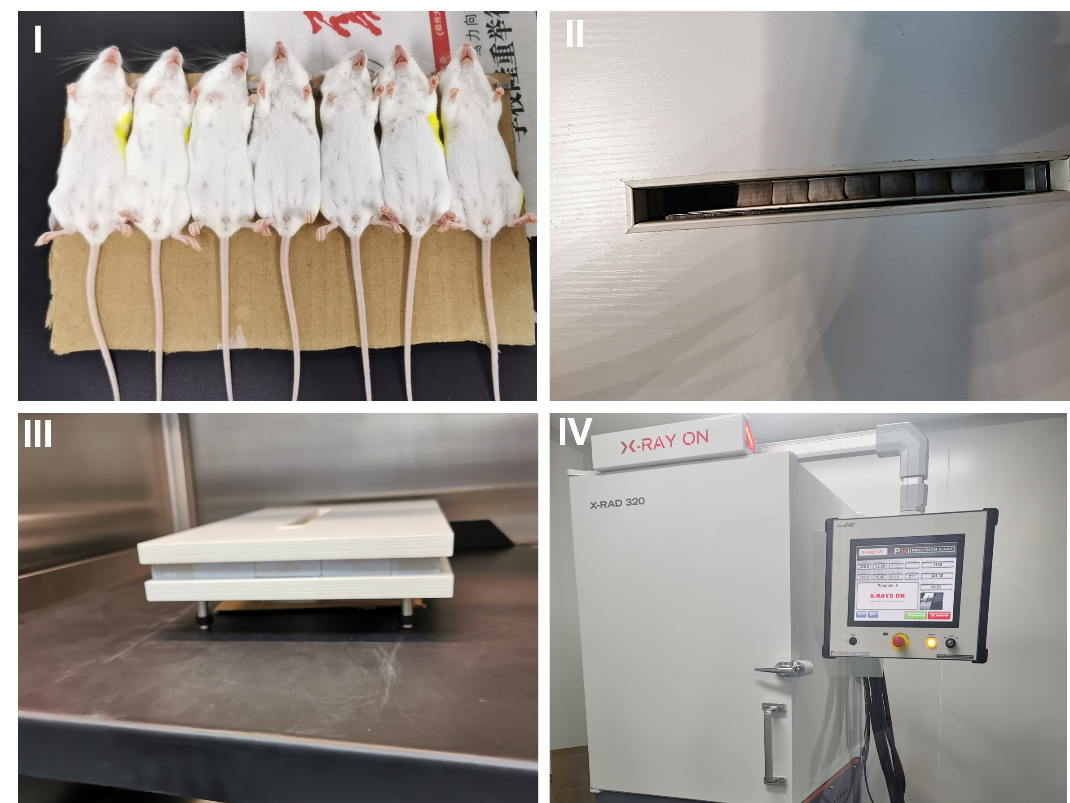


Figure S5. Radiation process of BALB/c mice.


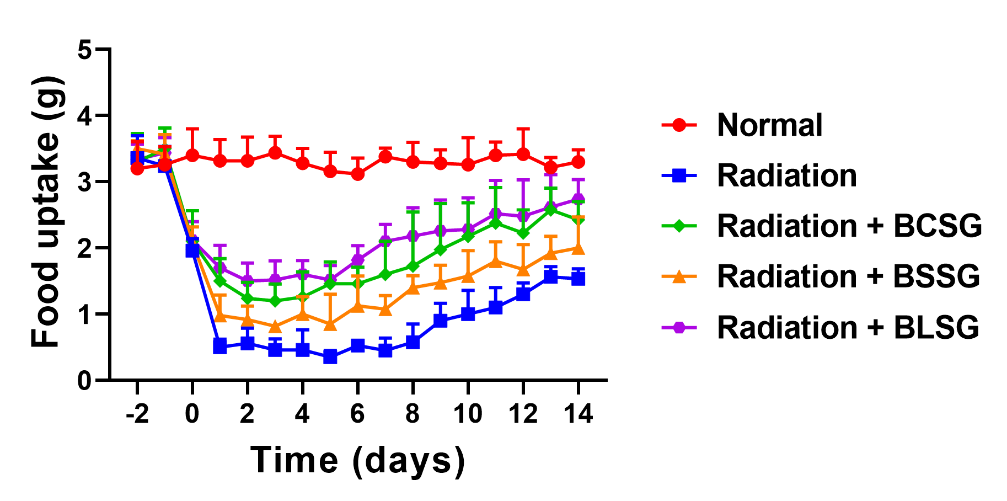


Figure S6. Food uptake chart mice during the period of treatment. Data are presented as mean ± SD (n = 5). p values were calculated by two-way ANOVA with a Tukey post-hoc test (**p < 0.01 and ***p < 0.001).


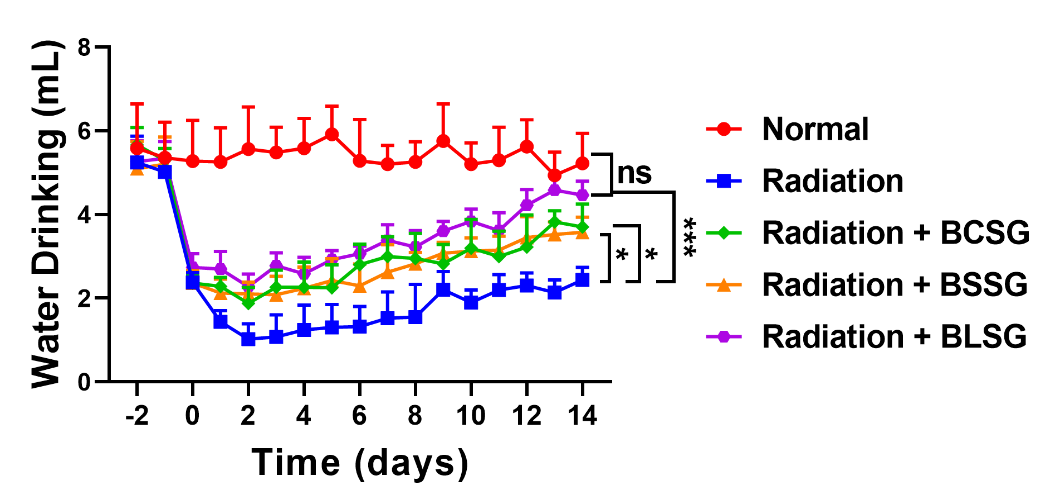


Figure S7. Water drinking chart of mice during the period of treatment. Data are presented as mean ± SD (n = 5). p values were calculated by two-way ANOVA with a Tukey post-hoc test (*p < 0.05 and ***p < 0.001).


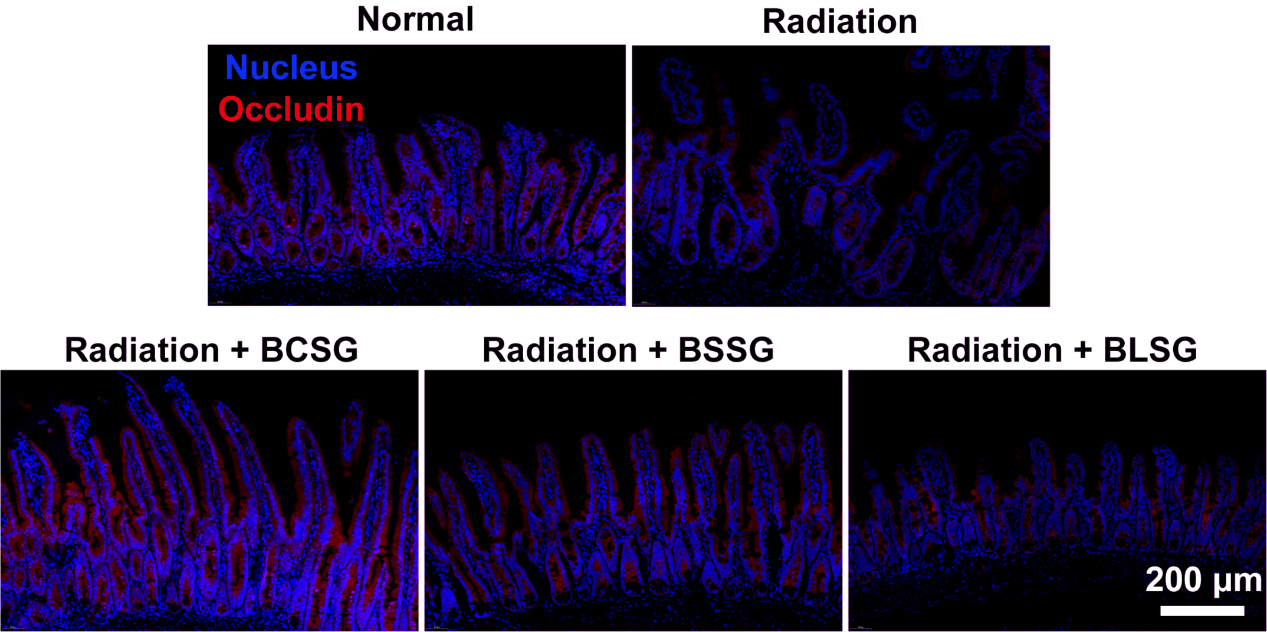


Figure S8. Occludin expression at 10th day.


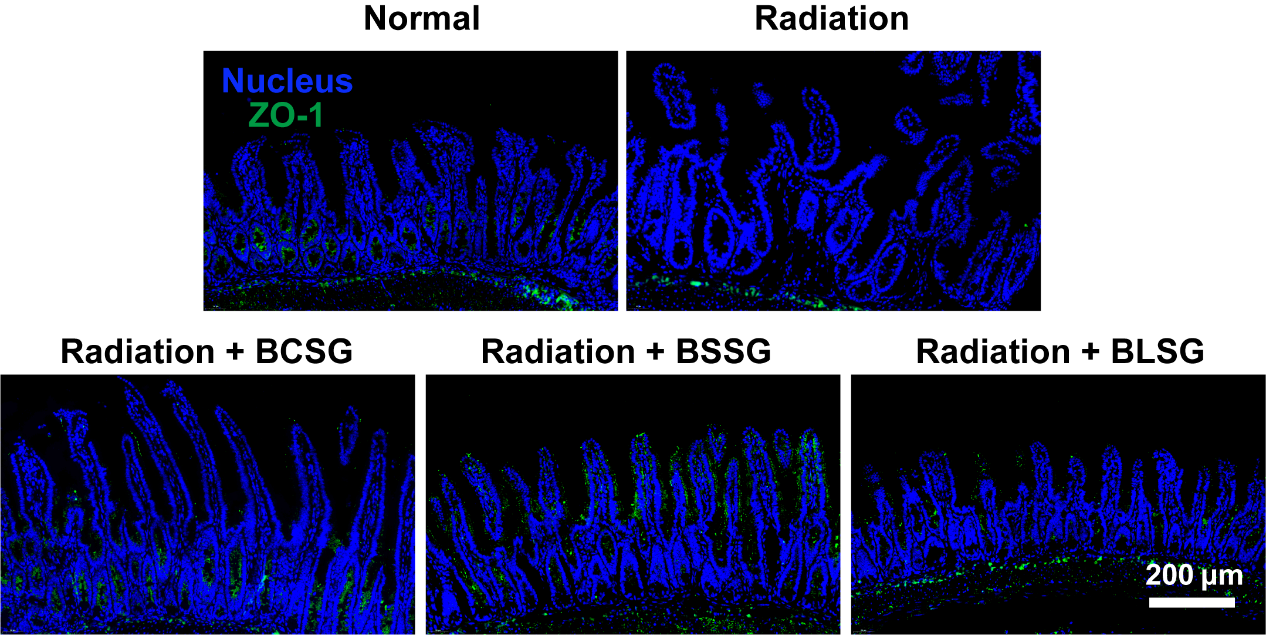


Figure S9. ZO-1 expression at 10th day.


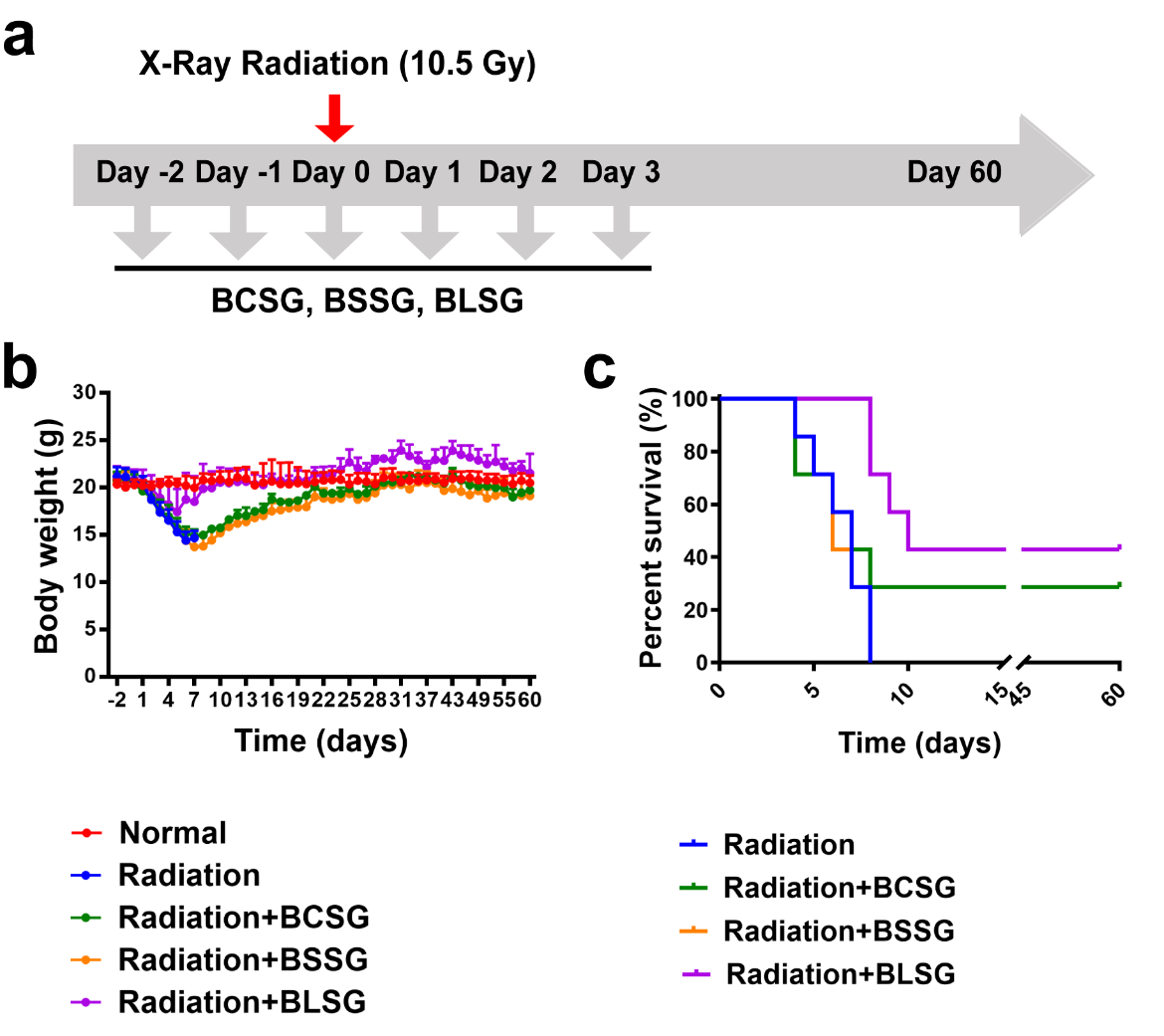


Figure S10. (a) Schematic illustration of the experiment protocol. (b) The body weight changes. (c) Survival curves of mice. Data are presented as mean ± SD (n = 7)


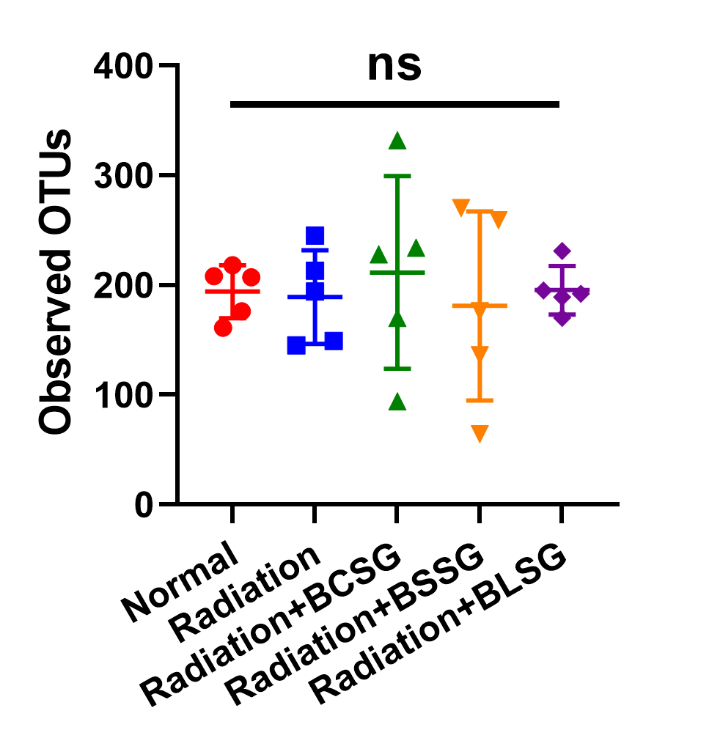


Figure S11. Coverage diversity index. Data are presented as mean ± SD (n = 5). p values were calculated by one-way ANOVA with a Tukey post-hoc test.


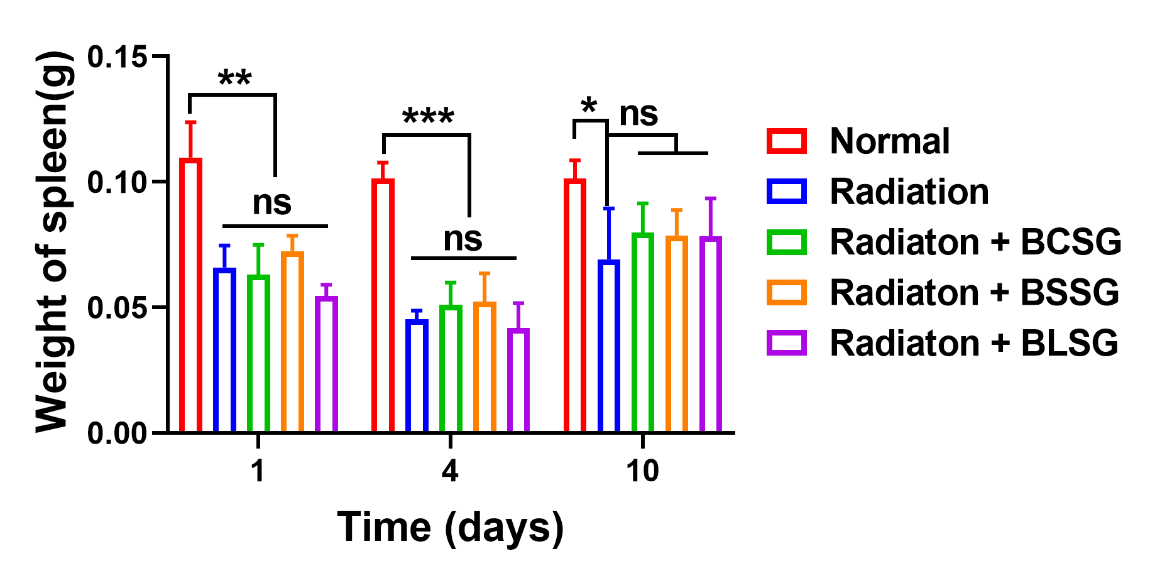


Figure S12. The spleen weight changes in mice. The data show means + SD (n = 5). p values were calculated by two-way ANOVA with a Tukey post-hoc test (*p < 0.05, **p < 0.01 and ***p < 0.001)


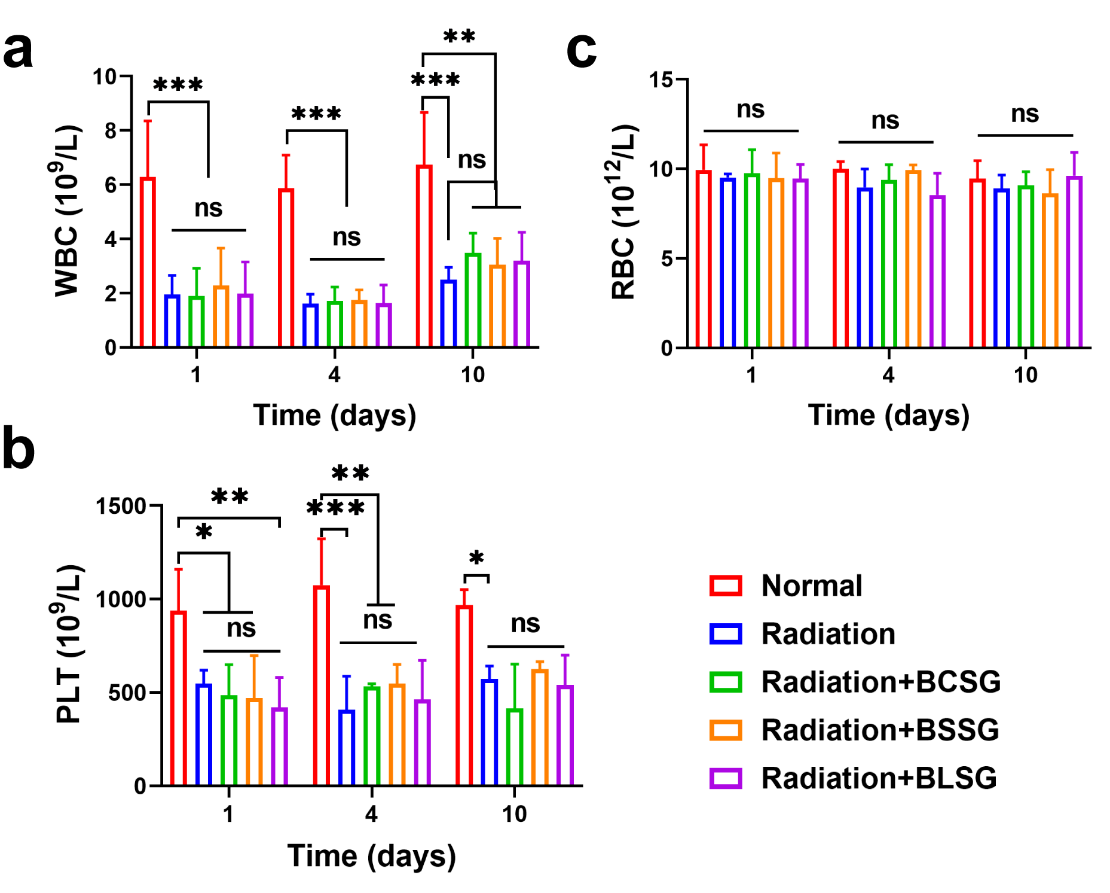


Figure S13. The blood routine examination at different time point. Data are presented as mean ± SD (n = 5). p values were calculated by two-way ANOVA with a Tukey post-hoc test (*p < 0.05, **p < 0.01 and ***p < 0.001).

Table S1. Elements content of BCSG, BSSG and BLSG.

|  | BCSG | BSSG | BLSG |
| --- | --- | --- | --- |
| Carbon | 57.33 | 81.03 | 45.68 |
| Nitrogen | 13.14 | 6.03 | 2.12 |
| Oxygen | 25.35 | 10.24 | 23.54 |
| Calcium | 1.19 | 0.51 | 6.33 |
| Manganese | 0.96 | 0.18 | 6.1 |
| Phosphorus | 1.96 | 1.30 | 6.41 |
| Sulfur | 0.06 | 0.70 | 8.36 |
| Zinc | 0 | 0 | 1.48 |
